# A microfluidic platform for highly parallel bite by bite profiling of mosquito-borne pathogen transmission

**DOI:** 10.1038/s41467-021-26300-0

**Published:** 2021-10-14

**Authors:** Shailabh Kumar, Felix J. H. Hol, Sujit Pujhari, Clayton Ellington, Haripriya Vaidehi Narayanan, Hongquan Li, Jason L. Rasgon, Manu Prakash

**Affiliations:** 1 Department of Bioengineering, Stanford University, Stanford, CA USA; 2 Insect Virus Interactions Unit, Department of Virology, Institut Pasteur, UMR2000, CNRS, Paris, France; 3 Center for Research and Interdisciplinarity, U1284 INSERM, Université de Paris, Paris, France; 4 Department of Entomology, The Center for Infectious Disease Dynamics, and the Huck Institutes of The Life Sciences, The Pennsylvania State University, University Park, PA USA; 5 Department of Pharmacology, Physiology and Neuroscience, University of South Carolina School of Medicine, Columbia, SC USA; 6 Department of Electrical Engineering, Stanford University, Stanford, CA USA; 7 Woods Institute for the Environment, Stanford University, Stanford, CA USA

**Keywords:** High-throughput screening, Lab-on-a-chip, Ecological epidemiology

## Abstract

Mosquito bites transmit a number of pathogens via salivary droplets deposited during blood-feeding, resulting in potentially fatal diseases. Little is known about the genomic content of these nanodroplets, including the transmission dynamics of live pathogens. Here we introduce Vectorchip, a low-cost, scalable microfluidic platform enabling high-throughput molecular interrogation of individual mosquito bites. We introduce an ultra-thin PDMS membrane which acts as a biting interface to arrays of micro-wells. Freely-behaving mosquitoes deposit saliva droplets by biting into these micro-wells. By modulating membrane thickness, we observe species-dependent differences in mosquito biting capacity, utilizable for selective sample collection. We demonstrate RT-PCR and focus-forming assays on-chip to detect mosquito DNA, Zika virus RNA, as well as quantify infectious Mayaro virus particles transmitted from single mosquito bites. The Vectorchip presents a promising approach for single-bite-resolution laboratory and field characterization of vector-pathogen communities, and could serve as a powerful early warning sentinel for mosquito-borne diseases.

## Abstract (web summary)

High-throughput molecular surveillance of mosquitoes carrying dangerous pathogens is currently challenging. Here the authors present Vectorchip, a low-cost microfluidic platform enabling multiplexed detection of mosquito DNA, viral RNA and infectious viral particles at single bite resolution.

## Introduction

Mosquito-borne diseases including malaria, dengue fever, chikungunya, West Nile encephalitis, Japanese encephalitis, and Zika fever afflict more than 300 million individuals every year, resulting in more than 500,000 deaths^1^. As a result of increasing environmental pressures and human migration, mosquitoes and diseases propagated by them are expected to increase their geographical range, placing more than 50% of the world’s human population at risk^2,3^. To curb this crisis, we urgently need new tools to expand our understanding of mosquito-pathogen communities and transmission mechanics. In addition, expanding the scale of surveillance of medically relevant mosquito species and associated pathogens is critical for the early deployment of informed preventative measures in communities^4,5^.

Molecular analysis of genomic material or proteins remains the gold standard for the detection and study of mosquito-pathogen communities in various settings. In order to obtain molecular samples for field ecology, mosquitoes are typically collected through different modalities of traps^6,7^ or via human landing catches, which remain an ethically questionable method for gathering data^8–10^. These resource-intensive sample collection strategies commonly suffer from a limited scale of operation. As a result, severe undersampling of mosquito and pathogen populations remains a major concern around the globe^11^. In addition, molecular analysis of mosquitoes in the lab or field is usually performed using either (i) whole body sampling of mosquitoes by homogenizing them^12^, (ii) analysis of mosquito saliva after extraction of salivary glands^13^, or (iii) forced mosquito salivation^14^ to determine the prevalence of pathogens in mosquito populations. In addition to requiring sample preparation of mosquitoes, these methods do not represent actual biting events thus providing limited information about the transmission dynamics such as the infectious viral load of a bite. More recently, alternative modes of sample collection exploiting e.g., sugar feeding or excreta have been proposed^15–19^. Although practical, these methods do not simulate biting events and typically cannot differentiate between pathogens in saliva versus the mosquito gut and as a consequence provide limited data regarding pathogen transmission. An important limitation of commonly used methods is that they only provide population level (i.e., pooled) information with a limited scope of improvement in resolution, and typically do not quantify live viruses. A significant gap currently exists in our knowledge, which requires novel tools to (i) quantitatively track the dynamics of pathogen transmission directly from single mosquito bites, and (ii) improve the throughput of molecular profiling of mosquito populations to boost surveillance strategies.

High-throughput molecular interrogation of saliva from mosquito bites can provide a new window into the dynamics of vector-borne disease transmission. Quantification of the genomic content of salivary droplets and pathogen transmission directly from single mosquito bites can help shed light on the dynamics of transmission at bite-by-bite resolution^20^. At the same time, parallel profiling of multiple biting events can lead to highly accelerated sampling of large vector populations in the field. Microfluidic technologies have revolutionized biological discovery via high-throughput approaches^21,22^, but are yet to be developed specifically for the interrogation of mosquito-borne pathogen transmission. Currently, an important barrier to developing such microfluidic methods is the lack of a suitable vector-device interface. Membrane materials such as parafilm and animal casings typically used to interface with mosquitoes^23–25^ exhibit poor thermal and chemical compatibility. As a result, these materials have been difficult to integrate with protocols for nucleic acid amplification, cell culture, and microfluidic device manufacturing. Therefore, it is imperative to look for materials, device architectures, and protocols that can change the landscape of vector-pathogen diagnostics.

In this work, we demonstrate Vectorchip—a microfluidic platform for molecular diagnostics of salivary droplets deriving from individual mosquito bites allowing on-chip detection of mosquito DNA, viral RNA, and infectious viral particles. This is enabled by a soft lithography-based fabrication strategy, resulting in reaction-ready, skin-mimic polydimethylsiloxane (PDMS) membranes which allow mosquitoes to bite and feed through them. We investigate the biting of various mosquito species using this chip, discovering biomechanical differences in biting capacity which can be used to create species-selective feeding barriers. Saliva from the biting activity of mosquitoes on Vectorchip is autonomously collected in individually isolated reaction chambers. Nucleic acid amplification assays conducted on-chip indicate that mosquito DNA and viral RNA are released during these biting events enabling identification of both the vector and pathogen species. Finally, we show direct quantification of live, infectious viral particles from mosquito bites using focus forming assays (FFA) on chip, enabling interrogation of pathogen transmission through direct profiling of individual mosquito bites at high throughput.

## Results

### Device design and fabrication

**Fig. 1:**
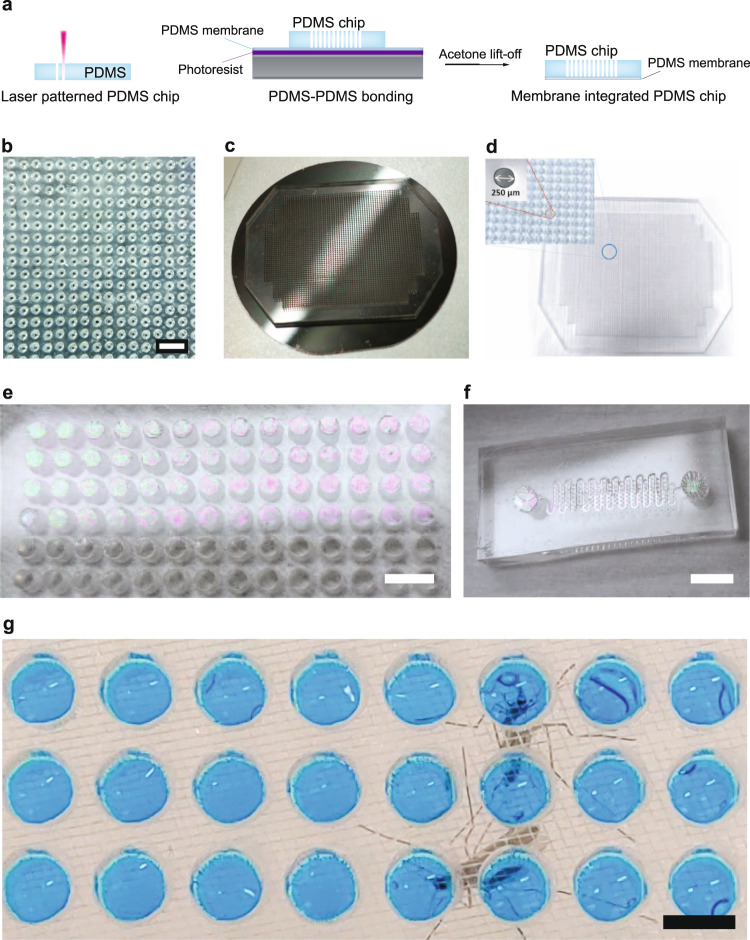
Fabrication of Vectorchip for collection of mosquito saliva and molecular assays. **a** Schematic showing the fabrication process of Vectorchip. Laser patterning was used to obtain through-holes in blocks of PDMS. A thin sacrificial layer of photoresist (Microposit™ S1813 G2, DOW Inc., USA) was spread onto a silicon wafer followed by spin-coating a thin film of PDMS on the wafer surface. The laser-patterned body of the chip was then bonded to the thin film of PDMS using plasma-activated PDMS-PDMS bonding. Membrane-bonded chips were obtained by placing the wafer in an acetone bath, where the sacrificial layer of resist was dissolved. **b** A PDMS block with laser-generated through-holes (diameter 250 μm). **c** A PDMS chip bonded to a 1.6 μm thin PDMS membrane on a 4-inch silcon wafer. **d** A Vectorchip with around 3000 wells (dia: 250 μm) is shown. **e** Chip with well diameter 1.75 mm and a visible PDMS membrane (thickness 1.6 μm). **f** Microfluidic channel-integrated chips with a PDMS membrane on top. **g** These wells can be loaded with feeding solution, reaction reagents, and can store mosquito saliva after biting assays. Wells loaded with feeding media (10% sucrose laced with blue food color) shown in the image. Scale bars represent 1 mm in (**b**), 5 mm in (**e**), and 2 mm in (**f**, **g**).

Vectorchips with integrated PDMS elastomer membranes were fabricated using a combination of laser patterning and soft lithography. We selected PDMS as the material of choice due to its low cost, biocompatibility, optical transparency, and chemical stability. PDMS-based devices can be fabricated using reproducible and scalable fabrication techniques^26^. Such devices have routinely been used for molecular analysis of small sample volumes using microfluidics^27^, and are compatible with molecular assays and cell cultures^21,22^. We used laser patterning to create open-ended arrayed compartments (3−4 mm deep) to capture saliva droplets from mosquito bites in PDMS micro-wells (Fig. 1a–d). In order to obtain ultra-thin PDMS films that the mosquito mouth parts can pierce, we utilized spin-coating of uncured PDMS over a flat silicon surface. We obtained membrane-integrated devices after plasma-induced PDMS-PDMS bonding of the laser-patterned array with the PDMS film (Fig. 1e). Detailed steps describing chip fabrication are included in the methods section below. The versatile fabrication process can provide user-defined variation in the size and density of the individual compartments. We were able to fabricate chips with hole diameters ranging from 150 μm to 1 cm (Supplementary Fig. 1) showing the scales of operation for the laser-ablation process while maintaining membrane stability. Additionally, the membrane can be easily integrated with fluidic networks for direct interfacing with mosquito bites, enabling assays involving on-chip fluid exchange (Fig. 1f). The microfluidic compartments on the chips can hold feeding media such as blood or sugar water (Fig. 1g), collect saliva during biting events, and act as isolated reaction chambers for molecular assays.

### Mosquito-chip interactions

**Fig. 2:**
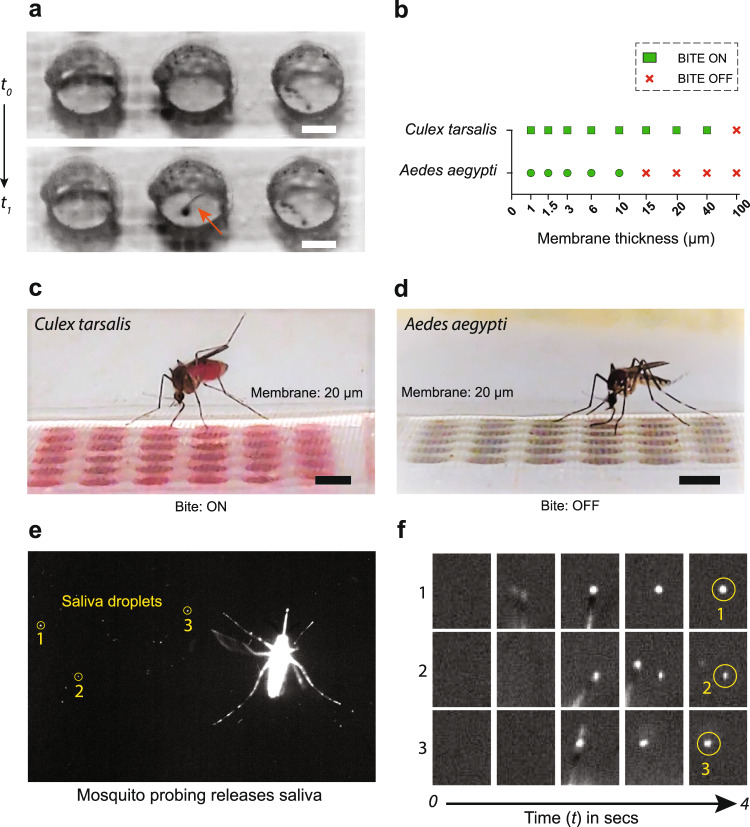
Mosquito biting on Vectorchip. **a** Stylet entry through a PDMS membrane for an *Aedes aegypti* female. **b** Mosquito feeding success as a function of membrane thickness for two mosquito species—*Aedes aegypti* and *Culex tarsalis*. We demonstrate that by changing the membrane thickness we can turn biting on or off. Biting off is defined as when no mosquito in the cage was able to feed from the chip in a duration of 30 min. The feeding assays were repeated at least three times (*n* = 3) at every given membrane thickness to confirm the ability of mosquitoes to feed through the membrane. **c** A *Culex tarsalis* female mosquito biting through a 20 *μ*m thick PDMS membrane. **d** Bending of the proboscis observed for an *Aedes aegypti* female, indicating failure in biting through the 20 μm membrane. **e** Fluorescent salivary droplets expectorated by an *Aedes aegypti* mosquito while probing. These mosquitoes were fed on rhodamine-laced sugar water resulting in fluorescent saliva. Three salivary droplets (1, 2, and 3) are encircled. **f** Timelapse images show a magnified view of fluorescent salivary droplet deposition (droplets numbered 1, 2, and 3 as shown in panel (**e**) over a period of 4 s). Scale bars represent 500 μm in (**a**) and 2 mm in (**c**, **d**).

Next, we examined the ability of mosquitoes to pierce through the PDMS membranes. Mosquitoes were attracted to the chips using heat as a guiding cue although several other baiting methods could be used. We placed a camera above the chip and observed stylet insertion through 1.6 μm thick PDMS membranes (Fig. 2a, Supplementary Movies 1 and 2). Abdominal engorgement in mosquitoes was observed after biting, indicating that mosquitoes can successfully feed through these membranes. Four species of mosquitoes were tested (*Aedes aegypti, Aedes albopictus, Culex tarsalis, Culex quinquefasciatus*) and all demonstrated successful probing and feeding through 1.6 μm thick PDMS membranes.

We also examined whether the membrane thicknesses can be engineered to selectively prevent the biting of some species while letting other species feed through the barrier. We loaded the laser-patterned microfluidic compartments with blood and warmed the chips to attract mosquitoes (Supplementary Fig. 2 and Supplementary Movie 3). We considered biting to be off when none of the mosquitoes in the cage demonstrated abdominal engorgement within 30 min since the start of the experiment. Tests were performed with *Aedes aegypti* (~45 mosquitoes per cage) or *Culex tarsalis* (~25 mosquitoes per cage) while varying the thickness of the PDMS membrane from 900 nm to 100 μm. We observed significant differences in the biting ability of the two species, where complete inhibition of feeding by *Aedes aegypti* was observed with membrane thickness at or greater than 15 μm (Supplementary Movies 4 and 5). In contrast, complete inhibition of feeding by *Culex tarsalis* was only observed for membranes that were 100 μm thick (Fig. 2b). This remarkable difference in the biting capacity of these two species has not been reported earlier. While some studies have focused on understanding the biting mechanics of a single mosquito species (*Aedes aegypti* or *Aedes albopictus*)^28,29^, we are not aware of any study which quantifies the differences in biting strength between different species and the evolutionary or biomechanical differences involved in this variation. Evolutionary pressure on mosquitoes biting different hosts could generate great variability of biting strength in mosquito species, which may be needed to probe different skin thickness. This intriguing biomechanical phenomenon can be advantageous for deploying devices where membranes of desired thickness can act as species-selective filters, providing a means to better track highly relevant species and pathogens in the field.

In order to extract molecular information from mosquito bites, we exploited the release of saliva during probing and biting events. The volume of saliva expectorated by mosquitoes in oil has been reported previously to be around 0.03 nL/min for *Culex pipiens*^30^, and 6.8 nL in approximately an hour for *Aedes aegypti*^31^ using a forced salivation method. *Aedes aegypti* have been estimated to salivate around 4.7 nL during blood feeding events^32^. We demonstrate that feeding rhodamine-labeled sugar water to mosquitoes renders their saliva fluorescent such that the release of saliva droplets can be directly visualized using fluorescence imaging (Fig. 2e and Supplementary Movie 6). Time sequences (Fig. 2f) show magnified images of three locations on a membrane surface where short probing events result in the deposition of fluorescent salivary droplets. We estimate that the volume of salivary droplets expectorated during this process is approximately 0.66 nL (Supplementary Fig. 3). This calculated mean volume is considerably lower compared to the volume associated with blood-feeding events reported earlier^32^; however, this discrepancy likely reflects differences between probing events such as observed in Fig. 2e, f and full feeding events as reported by Devine et al.^32^. The deposited salivary droplets harbor several biomarkers in the form of mosquito salivary proteins^33^, active pathogens, mosquito cells, and/or nucleic acids that can be used for their identification^14,20,34^.

### Distribution of bites on chip

An important consideration for molecular surveillance of mosquitoes is the granularity at which the population is sampled. Methods can range from pooling strategies with low coverage where samples are pooled together at the population scale, to the individual collection of saliva from mosquitoes while recording species identity. Vectorchip potentially provides an adjustable method between these two strategies by increasing or decreasing the density of bite wells presented to a population of mosquitoes.

**Fig. 3:**
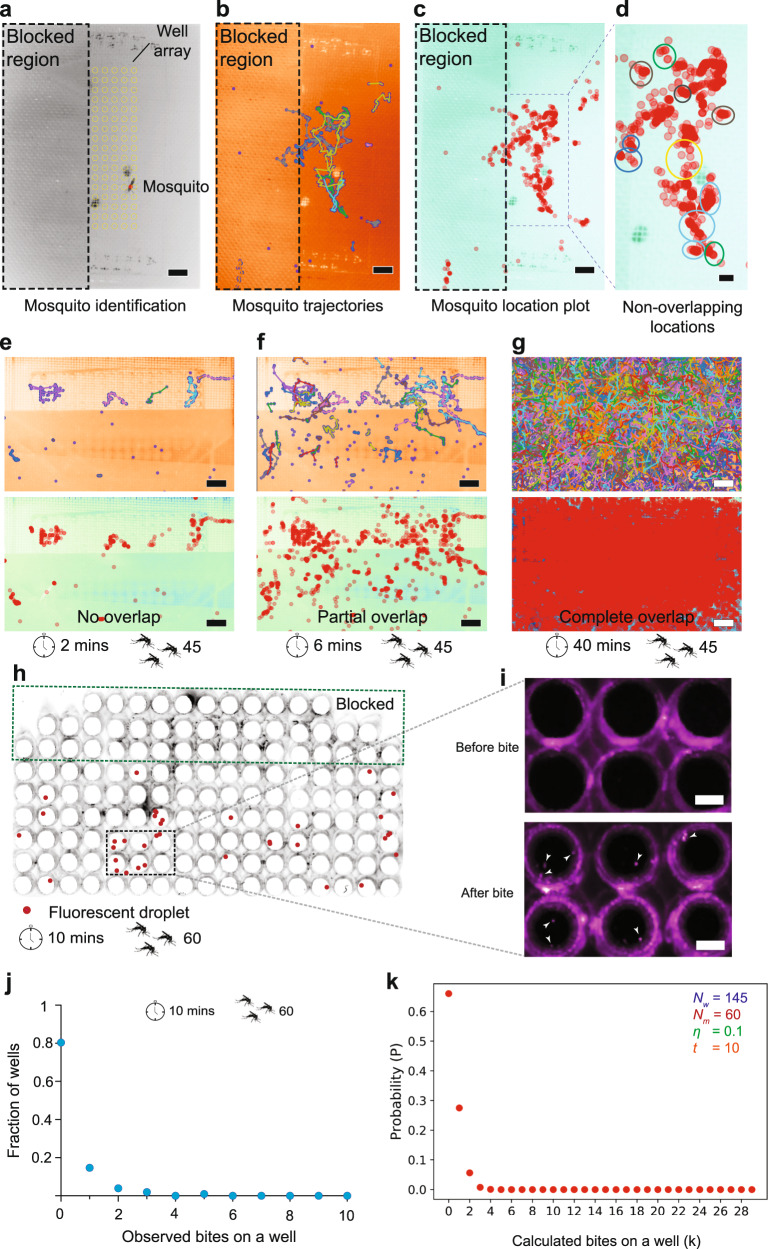
Tracking mosquito activity on chip. **a** Image recognition software was used to identify and track mosquitoes on the chip. Wells are highlighted in yellow. **b** Trajectories of mosquito movement can be plotted and indicate the overlap between different movement tracks. **c** Location plot of mosquitoes on the chip are indicated by red circles. Regions with overlapping red circles are darker in color. **d** Trajectory and location plot can be used to identify regions with individual mosquito locomotion activity as compared to regions with more crowding. **e**−**g** Varying experimental parameters (symbols indicate the duration of experiments and number of mosquitoes) can be used to obtain either individual mosquito plots or population data. Three cases are discussed with **e** no overlap between multiple probing trajectories, **f** partial overlap, and **g** complete overlap. **h** Representative image for a chip where the presence of fluorescent droplets on wells has been indicated by a red dot. **i** Zoomed-in fluorescent image of the same chip showing wells with single, or multiple droplets. **j** Number of bites per well were calculated by counting fluorescent droplets deposited on the wells after biting events. **k** We defined the number of mosquitoes = *N*_*m*_, the number of wells = *N*_*w*_, probing frequency = *η*, and time = *t*. The probability of obtaining ‘*k*’ bites on a well for the parameters (*N*_*m*_, *N*_*w*_, *η*, *t*) utilized in (**h**−**j**) has been shown. Scale bars represent 5 mm in (**a**−**c**, **e**−**g**), 2 mm in (**d**), and 1 mm in (**i**).

We first analyzed the distribution of bites on Vectorchip by tracking mosquito activity during biting assays. In order to perform sucrose biting assays for molecular diagnostics, we filled the wells with 10% sucrose, and covered the open side of the chip with a glass slide. A resistor that acts as a heat source was placed atop the glass slide and the chip-resistor assembly was placed on top of the mesh ceiling of a mosquito cage. A part of the chip (50 percent of the total chip area) was protected by paper tape such that mosquitoes could not bite into that section of the device. The area of the chip protected by tape served as negative control for the downstream molecular assays. A camera (Raspberry Pi 3 camera module V2.1) was placed on the bottom of the cage to track and record individual mosquito activity on chip (Supplementary Fig. 4). Image recognition and mosquito tracking algorithms have been described by us previously^25^, where we used computer vision to analyze image sequences and robustly track the presence, movement, and biting behavior of individual mosquitoes at high resolution. In this study, we have used an imaging setup where the camera is attached to the bottom of the cage (Supplementary Fig. 4a). The obtained image datasets reveal mosquito trajectories on the chip as well as areas with prolonged interactions (Fig. 3a–d, Supplementary Fig. 4, and Supplementary Movie 7). Unique trajectories are identified as movements performed by an individual mosquito while in the field of view (e.g., landing, exploration, biting, and take-off by a single mosquito would constitute a trajectory). The movement of mosquitoes on a chip is shown in Fig. 3e–g and Supplementary Fig. 4. Depending on the duration of mosquito-chip interactions, multiple trajectories do not overlap (Fig. 3e), partially overlap (Fig. 3f), or completely overlap (Fig. 3g). Trajectory plots can indicate the likelihood that a set of wells has been probed by more than one mosquito. These results indicate that by modulating the time of interaction between the mosquito population and the chip, the user can control the degree of probing experienced by the chip. As an example, a chip with no overlap between trajectories should exclusively contain wells probed by single mosquitoes. Tracking mosquito movement on chip was used to locate trajectories and thus regions with unique mosquito presence.

While tracking provided macro-scale information about mosquito position and activity, we also utilized the release of fluorescent salivary droplets deposited by biting mosquitoes to resolve mosquito probing events at higher resolution. We quantified the number of fluorescent spots seen in every well of the PDMS chip by using fluorescent scans of the chip before and after biting (Fig. 3h). No spots were observed in the negative control region where access was blocked to mosquitoes, while several fluorescent spots observed on the open areas of the chip indicate the wells which have been probed. The images show the presence of wells with multiple bite marks, as well as single bite marks.

We further examined the design parameters used to build Vectorchip that can dictate the resolution of this sampling strategy. The primary determinants of how often a single mosquito will bite a specific well are dependent on multiple factors, including the number of mosquitoes in the cage, the time mosquitoes have access to the chip and biting behavior (e.g., frequency), and the size and number of wells on the chip. Other additional factors like the feeding media may affect the number of biting events performed by a female mosquito. In this study, we focused on sucrose as the feeding media since it is a diet source that does not inhibit polymerase chain reaction (PCR) assays. We considered a simple mathematical model to arrive at a first approximation for the distribution of bites on a chip. In this model, we made a simple assumption that mosquitoes probe the chip at a fixed frequency (*η*). We also assumed that mosquito bites are random and independent events, since no spatial gradients have been created on the chip. Detailed analysis of this model can be found in Supplementary Section 1.

We defined the number of mosquitoes = *N*_*m*_, the number of wells = *N*_*w*_, and probing frequency = *η*, and estimated the probability (*P*) that a well receives *k* bites in given time *t* as (Supplementary Note 1):

1 $$P[k]=({N}_{m}\eta t)!/(k!\times ({N}_{m}\eta t-k)!)\times 1/{N}_{w}^{k}\times {(1-1/{N}_{w})}^{{N}_{m}\eta t-k}$$

This was approximated by the form (Supplementary Note 1):

2 $$P[k]\approx {{{{{{\mathrm{e}}}}}}}^{-{N}_{m}\eta t/{N}_{w}}\times {({N}_{m}\eta t/{N}_{w})}^{k}/k!$$

and represents a Poisson distribution of the form,

3 $$P[k]={{{{{{\mathrm{e}}}}}}}^{-\lambda }\times {(\lambda )}^{k}/k!$$

with the expected rate of events (i.e., bites per well), *λ* = *N*_*m*_*ηt*/*N*_*w*_. Probability distributions for the expected number of bites on a well were plotted while varying the number of mosquitoes, wells, and probing frequencies (Supplementary Fig. 5). The probing frequency of *Aedes aegypti* mosquitoes on a parafilm membrane has been estimated previously to be in the range of 0–1 bites per min^35^. Supplementary Fig. 5 shows a diverse phase space where a single bite per well is the most dominant outcome, i.e., *λ* was less than or equal to 1, when up to 50 mosquitoes interact with a 150-well Vectorchip for 15 min—in line with our experiments. In the context of a larger number of mosquitoes interacting with the chip, increasing the number of wells can ensure that the majority of wells will not get bitten more than once. We quantified bites on wells as indicated from fluorescent droplets (Supplementary Table 1), and compared it to the analytical model (Fig. 3j, k). The fitting parameter was *η* = 0.1 bites per minute, which is close to the range of probing frequencies reported recently^35^. With water as the feeding media and parafilm as the probing membrane, Jove et al. report that the majority of tested mosquitoes showed a probing frequency below 0.5 bites per minute^35^. While we observe and model slightly lower probing frequencies, this difference is possible due to the change in the membrane material from parafilm to PDMS, and other differences in the setup. The methods discussed above provide multiple ways to understand mosquito−chip interactions as well as help us define parameters to obtain data at single-bite resolution.

### PCR diagnostics on-chip

Having established that mosquitoes can bite through the membrane and deposit salivary droplets in microwells while biting on a Vectorchip, we analyzed these bites for their DNA and RNA content. Running PCR-based nucleic acid amplification directly on individual wells establishes a low-cost assay to detect bite content in a high-throughput manner. We utilized devices with a PDMS membrane thickness of 1.6 μm and well diameter of 1.75 mm to perform molecular analysis of salivary samples; providing 145 independent reactions per chip (within a chip area of 5 cm × 2.5 cm).

**Fig. 4:**
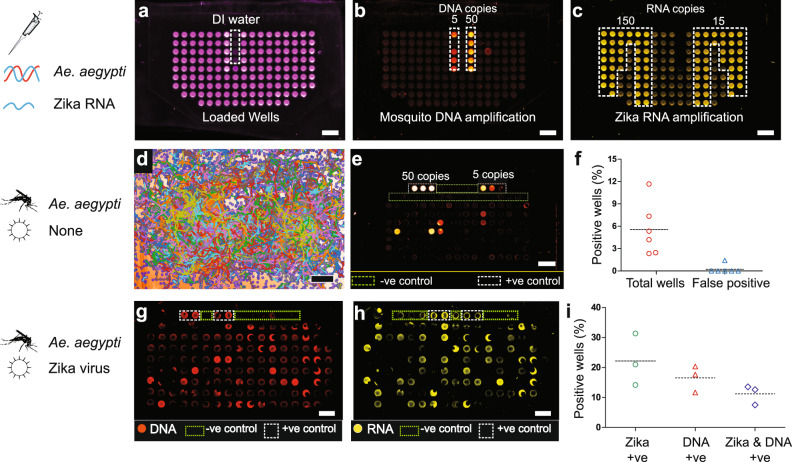
On-chip PCR for detection of mosquito and pathogen. The symbols indicate manually spiked assays, uninfected mosquito assays, and Zika infected mosquito assays from top to bottom. **a**–**c** A spiked assay with successful amplification of *Aedes aegypti* DNA and Zika RNA on chip. **a** Indicates wells filled with PCR mix, with ROX dye providing the background fluorescence. Fluorescent wells indicate amplification of **b** mosquito DNA, and **c** Zika RNA on chip. Assays were performed with uninfected mosquitoes where **d** tracking patterns were collected and demonstrate extensive translocation activity on the chip. **e** PCR shows detection of mosquito DNA on the chip directly from biting. **f** Percentage of wells available for biting on Vectorchip that display a positive PCR outcome. The PCR assay was repeated 6 times on different chips (*n* = 6). The false positive rate (amplification in wells that were covered by tape) was close to zero (0.23%). Assays performed with Zika infected mosquitoes indicate the presence of both **g** *Aedes aegypti* DNA and **h** Zika virus RNA after bites on chip. **i** Percentage of wells available for biting that show a positive signature for Zika RNA, mosquito DNA or both. The PCR assay was repeated 3 times on different chips (*n* = 3). Scale bars represent 5 mm in (**a**−**e**) and 3 mm in (**g**, **h**). Source data are provided as a Source Data file.

Detection of mosquito mitochondrial DNA (mtDNA) and RNA from viruses in saliva was performed using on-chip PCR with end-point fluorescence readout. A detailed protocol for performing PCR in Vectorchip is provided in the Methods section and Supporting Information file (Supplementary Fig. 6). We tested the efficiency of PCR amplification in Vectorchip by manually loading a known concentration of DNA and RNA into the reaction wells (Fig. 4a–c). We performed the PCR for 42 cycles and detect DNA amplification from approximately 5 DNA copies in 4 μL of reaction mix (~1 copy/μL) (Fig. 4b). Simultaneously detection of Zika virus RNA using reverse transcription-PCR (RT-PCR), was successfully demonstrated from as low as 15 RNA copies in 4 μL of reaction mix (Fig. 4c). PCR reactions were also performed in 96-well plates to test if amplification accuracy and sensitivity were similar as compared to Vectorchips. Both qPCR amplification curves and end point fluorescence information was obtained from the well plates. The 96-well plate reactions were performed in DI water and also in the presence of dried sucrose (10%) to ensure that the amplification response provided a good comparison to the Vectorchip-based reactions using manually spiked concentrations, and to test if the presence of sucrose would inhibit the reactions (see Supplementary Fig. 7a, b).

Vectorchip provides an opportunity for tracking both pathogens in salivary bites, as well as identifying host species by detecting DNA from the host biting the well. Mosquito DNA has previously been reported in salivary droplets deposited by feeding *Culex* mosquitoes^20^. Utilizing uninfected *Aedes aegypti* mosquitoes, we performed PCR assays on-chip to detect the presence of mosquito DNA in deposited saliva droplets. Figure 4d, e shows the tracking data and PCR detection of mtDNA for a chip, which was placed on a cage with 75 uninfected *Aedes aegypti* females for 45 min. Areas highlighted in green were protected by paper tape such that mosquitoes could not probe them and serve as negative controls. The tracking data obtained from the chip indicate significant activity of the mosquitoes on-chip as represented by several overlapping trajectories. PCR data confirms that mosquito DNA indeed can be detected in probed wells. Multiple experiments indicate that a subset (3−12%, *n* = 6) of the wells where mosquitoes are active test positive for the presence of mosquito DNA (Fig. 4f). The rate of false positives obtained from wells not accessible to mosquitoes was very close to zero (0.23%, *n* = 6). In order to further validate the rate of detection of mosquito DNA on Vectorchips, we performed a biting assay using a 96 well plate loaded with agarose and covered with a parafilm membrane. Agarose gel was used as feeding media during this test, to minimize the risk of cross-contamination between wells during the peeling off of the parafilm membrane (details in “Methods” section). We recorded the tracking and DNA amplification response from uninfected mosquito bites on the well plate (Supplementary Fig. 7c, d). Approximately 15% of the wells where mosquitoes were active tested positive for mosquito DNA, which is close to the fractions observed on Vectorchips. Since mosquito DNA can only be detected in a subset of wells showing mosquito activity, this suggests that the presence of detectable levels of mosquito DNA in saliva is a noisy physiological process, likely dependent on multiple variables such as duration of feeding and time of last bite. It is furthermore important to note that our tracking algorithm only detects the presence of mosquitoes, but does not provide information regarding if a well was bitten or not.

Next, we utilized the device to perform assays on infected mosquitoes, for the detection of both mosquito species and deposited pathogens. Figure 4h shows results obtained when *Aedes aegypti* infected with Zika virus (ZIKV)^36^ interacted with the chip for 20 min. We subsequently performed RT-PCR on the chip and observed that *Aedes aegypti* mtDNA and ZIKV RNA could be detected in 24/118 and 37/118 wells, respectively. We summarize the detection rates of mosquito DNA and ZIKV RNA for three assays performed with ZIKV-infected mosquitoes in Fig. 4i. Interestingly, not all wells positive for ZIKV RNA showed positive for mosquito DNA and vice versa, highlighting the stochastic genetic composition of bite-derived nanoliter saliva droplets. A higher fraction of wells (mean ~ 22%, *n* = 3) showed positive for ZIKV RNA as compared to mosquito mtDNA (mean ~ 17%, *n* = 3), indicating a higher prevalence of viral genomic material in mosquito saliva as compared to mosquito mtDNA. Detection of mosquito DNA and virus RNA in these assays demonstrates the capacity of this tool to identify mosquito and pathogen species directly from mosquito bites on-chip.

### Detection of infectious viral particles directly from bites

While PCR-based end-point assays are an important strategy for multiplexed detection of vectors and pathogens in various settings, they cannot determine the presence or concentration of infectious virus particles in bites. Detection of infectious pathogen load in mosquito bites is important to understand the vector competence (including potential and efficiency of pathogen transmission through bites) of mosquito-pathogen systems and its dependency on factors such as local climate^37^, mosquito species^38^, physiology^39^, and presence of endosymbionts (e.g., *Wolbachia*)^40^. Typical methods to measure vector competence rely on manual “forced salivation” of individual mosquitoes^12^, where the viral loads may differ from biting events and mosquitoes are sacrificed preventing time-course measurements. Sugar feeding on filter papers has also been utilized recently, and provides promising results towards longitudinal sampling of viral transmission by individual mosquitoes^20^. As compared to filter paper-based approaches, the Vectorchip uses an integrated PDMS membrane which can enable purely bite-based collection and avoid possible contamination from e.g., excretion events. Furthermore, our method improves the throughput of the assay as mosquitoes do not need to be individually housed and sampled. Finally, filter paper assays are reliant on sampling nucleic acids, and cannot perform live cell culture assays to detect infectious pathogens.

**Fig. 5:**
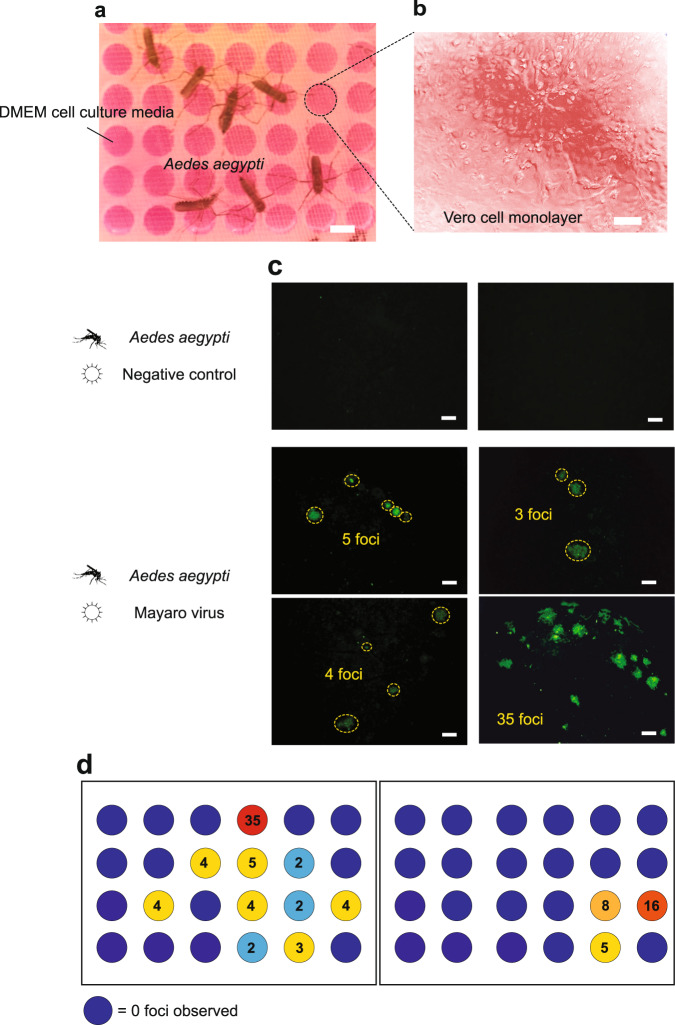
Quantifying active viral particles using focus-forming assays. Focus forming assays on Vectorchip can directly quantify the number of active viral particles in mosquito bites, without the need for isolation of individual mosquitoes and manual salivary extraction. **a** Image shows *Aedes aegypti* mosquitoes infected with Mayaro virus biting on Vectorchips filled with DMEM cell culture media. **b** A monolayer of vero cells was cultured on Vectorchip membrane. The formation of vero cell monolayers on membranes was verified on 5 independent chips (*n* = 5). **c** FFA performed on a Vectorchip. Symbols indicate wells in the top row which were bitten by mosquitoes with no infection (negative control), and rows of wells bitten by mosquitoes infected with Mayaro virus. The green channel shows fluorescence from an antibody against a viral envelope protein. Every green island represents infected cells likely to have resulted from an active viral particle. No active viral particles were seen in the control wells. FFA on Vectorchips was verified on 3 chips (*n* = 3) for both uninfected and Mayaro infected mosquitoes. **d** FFA formed on two chips and viral foci were counted using the antibody fluorescence. Scale bars represent 2 mm in (**a**), 30 μm in (**b**), and 100 μm in (**c**).

We tested the potential of Vectorchip to perform focus forming assays (FFA) and detect infectious viral particles directly as a result of mosquito bites on chip (Fig. 5). Vectorchip wells were filled with cell culture media (Dulbecco’s modified eagle medium (DMEM) supplemented with 10% fetal bovine serum (FBS). The efficiency of FFA on-chip was tested using manual spiking of viral particles into Vectorchip (Supplementary Fig. 8). In order to perform biting assays, *Aedes aegypti* mosquitoes were infected with Mayaro virus, an emerging zoonotic pathogen that causes a dengue-like disease^41^. The mosquitoes were able to bite through the membrane into the wells and transfer live Mayaro viral particles directly into the cell culture medium (Fig. 5a). These viral particles then infect a monolayer of vero cells cultured on the PDMS membrane. These infections can be identified via fluorescent antibodies specific to viral envelope proteins (Fig. 5c). Each fluorescent patch (focus) is attributed to an infection resulting from a single viral particle. Foci counted on two sample chips can be seen in Fig. 5d. No foci were visible from negative control samples. The distribution of viral foci on the chips indicates the heterogeneity of infectious particle dose in mosquito bites. Figure 5 shows that the Vectorchip supports the growth of a cell monolayer on the chip and can enable the direct collection of mosquito saliva through bites in cell culture media. Antibody assays can be used to directly quantify infectious viral particles in the wells. These results demonstrate the suitability of using the Vectorchip to probe the transmission of infectious viral particles.

Finally, we examined if the feeding media provided in Vectorchip can enable blood meal-mimic biting behavior through the addition of phagostimulants such as ATP, while maintaining accurate molecular analysis (Supplementary Note 2). We observed that the addition of 100 μM ATP, (which is a phagostimulant and used by mosquito sensory neurons to identify blood^35^), to the feeding media did not impact nucleic acid amplification reactions (Supplementary Fig. 9a) (*n* = 3). Interestingly, mosquitoes feeding on DMEM supplemented with 10% FBS and ATP (1 mM) led to a similar or higher number of abdominal engorgements in 45 min as compared to blood meals (*n* = 3) (Supplementary Fig. 9b). This is reasonable as DMEM (supplemented with FBS and ATP) contains a variety of the components used by mosquito sensory neurons to identify blood at relevant concentrations (Supplementary Note 2)^35^). In summary, our data indicate that Vectorchip can provide physiologically relevant mosquito feeding behavior, enabling accurate assessment of vector and pathogen identity through nucleic acid amplification reactions for field surveillance, as well as on-chip assessment of transmission of infectious viral particles in bites through focus forming assays.

## Discussion

We demonstrate a microfluidic device integrated with a PDMS “artificial skin” membrane for molecular diagnostics of individual mosquito bites on a chip. The fabrication scheme permits straightforward user-desired modifications in chip design, as well as more than two orders of magnitude variation in reaction chamber volume for performing molecular analysis. The elastomer membrane is compatible with scalable device fabrication, microfluidic integration, nucleic acid amplification assays, as well as cell culture assays, allowing multimodal interrogation of mosquito bites on chip. Interfacing mosquito bites with fluidic channels provides the potential of using this device with integrated vascular pathways or particle traps to study transport and subsequent infection dynamics of expectorated pathogens. As compared to previous methods, the Vectorchip uniquely allows quantitative interrogation of single mosquito bites on a chip, which may answer questions such as the genomic diversity in individual salivary droplets, transmission dynamics of pathogens in bites, and enable high throughput diagnostics of large mosquito populations at single bite resolution.

We tested our devices with four mosquito species and all were able to probe through ultrathin PDMS membranes (thickness 1.6 μm) demonstrating the ability of this device to interface with a variety of vectors. Furthermore, our fabrication strategy permits us to engineer the “skin characteristics” such as thickness and stiffness. We discover that different species of mosquito can display significant variation in their biting strength, the biomechanical basis for which remains an intriguing question. This variation in probing capacity provides an opportunity to perform mechanical species selection for on-chip sample collection by careful engineering of the membrane thickness and stiffness. This observation also provides opportunities towards examining the biting capacity in different mosquito species and if there exists an evolutionary relationship between biting ability and the preferred host species. The potential of this method further depends on the role that size differences between individual mosquitoes vs. species differences play in determining mosquito bite strength, which as of yet remains unknown requiring further studies.

We utilized an analytical Poisson model, tracking algorithms, as well as experimental analysis to dissect multiple variables determining the distribution of bites per well on the chip. We further used this analysis to control the variables in assays and obtain transmission data at single bite resolution. Combining high-throughput data collection with single bite analysis, the Vectorchip aims to significantly reduce the labor, time, and risk associated with capture, individual segregation, and sample extraction from individual mosquitoes as traditionally practiced. Reaction in miniaturized chambers reduces the cost of molecular assays—chips used in this report (1.75 mm diameter) performed PCR reactions at 30 ȼ-per reaction chamber which is approximately 5× lower than current single mosquito homogenization assays. We also fabricated further miniaturized device designs with smaller well diameters, which can provide a 100× reduction in individual reaction volumes lowering the cost per-assay.

We detected that biting mosquitoes release their DNA, which can be used to identify their species as well as targeted genetic mutations. Detection of these genetic signatures can be used for monitoring the spread of native or invasive mosquito species globally, insecticide resistance in mosquitoes, or tracking the distribution of gene-drive-carrying mosquitoes released at experimental sites^42^. Detection of pathogens was demonstrated using (i) RT-PCR assays for the presence of viral nucleic acids and (ii) FFA to quantify infectious viral particles directly from mosquito bites. PCR assays can enable low-cost sampling of pathogens in the field informing community action for disease prevention. Simultaneously, FFA results provide a window to dynamics of infection transmission directly through individual mosquito bites, which as of yet remains difficult to study.

Previous reports have indicated that mosquitoes can secrete varying volumes of saliva in different substrates (e.g., sucrose as compared to blood meals)^43^. We have also shown that the substrate can affect engorgement rates, where ATP-enriched cell culture media and blood media-enabled more engorgement as compared to sucrose solution or DI water. In spite of these differences, we believe that the sucrose solution utilized as a feeding medium in Vectorchips provides several advantages for field-based surveillance of mosquito-pathogen communities, due to ease of accessibility, application, and storage. Additionally, probing by mosquitoes has been demonstrated to be sufficient to expectorate saliva and release detectable pathogens for surveillance efforts^15,20^. Alternately, substrates such as blood (or blood meal mimics) promote a more physiologically relevant feeding response and might be used for accurate assessment of transmission of active viral pathogens in individual bites.

The distribution of pathogens in our environments is currently significantly undersampled. This contributes to dramatic consequences in the form of unexpected viral outbreaks. There is an urgent need for vector-pathogen sampling to shift from current resource-intensive practices to low-cost technologies that can perform large-scale surveillance. We hope tools like the Vectorchip will allow us in the future to completely eliminate human-landing catches. The Vectorchip provides a route for multimodal analysis of vector-pathogen dynamics both in lab and field including vector identification, biting behavior, and pathogen surveillance at high throughput and resolution. We anticipate that the Vectorchip can be utilized for vector and pathogen surveillance testing in field sites, analysis of pathogen transmission dynamics under diverse settings in the lab, study of vector biting behavior on chip, and can also be expanded to other biting vectors such as ticks and sandflies.

## Methods

### Chip fabrication

PDMS chips with thickness ~4 mm were prepared as the main body of the chip. Sylgard 184 base and cross linker (Dow Chemicals, USA) were mixed at a 10:1 ratio and cured at 75 ^∘^C on a flat heater surface. Laser ablation (60-Watt Mini CO_2_ laser, Epiloglaser, USA) was used to cut holes through the PDMS blocks with desired hole diameter and density. Debris produced by the laser ablation process was removed using 1 h sonication in acetone. The chips were then rinsed with DI water and dried.

PDMS membranes were prepared over single-side polished, 4-inch silicon wafers. A layer of positive photoresist (Microposit™ S1813 G2, DOW Inc., USA) was spun on the wafers to a thickness of around 2 μm (1500 rpm for 45 s). The wafer with the photoresist layer was softbaked at 80 ^∘^C for about 30 min. PDMS (Sylgard 184 kit with base and curing agent mixed at 10:1 ratio) was freshly prepared and spuncoat on top of the wafers. The thickness of the PDMS coat was controlled by varying the spin speed and time. In order to obtain a membrane with thickness close to 1.6 μm, PDMS was spun on the wafers at 6500 rpm for 10 min. Wafers with thin layers of PDMS were cured at 70 ^∘^C for at least 6 h. The thickness of the PDMS on the wafers was measured by cutting strips off of a silicon wafer and measuring the difference in height using a profilometer (KLA Tencor Alpha Step D500).

The main body of the chip and wafers with thin PDMS membranes were placed in a plasma cleaner and exposed to O_2_ plasma (80 W, 45 s, 2 sccm O_2_ flow). The PDMS chips were then placed in contact with the thin PDMS membranes to enable bonding of the membranes to the chips. The wafers were baked at 80 ^∘^C for around 2 h to improve the bonding strength. A razor blade was used to cut outlines around the chips, such that membrane-attached PDMS chips could detach from the wafers. The wafers were then placed in acetone for approximately 2–4 h. The sacrificial resist layer was dissolved in acetone and PDMS chips with integrated ultrathin membrane were separated from silicon wafers. The chips were then rinsed gently in DI water and dried in an oven.

### Biting assays

Vectorchips were hydrophilized by placing in a plasma cleaner (Harrick Plasma) at high power for 5 mins. In order to test the biting ability of mosquitoes, sugar water was removed from the cages approximately 18 h prior to the assays. Chips with varying membrane thickness were filled with defibrinated sheep blood (Hemostat laboratories, USA). The well diameter was kept constant at 1.75 mm. Chips were placed on mosquito cages for approximately 30 min, and blood-feeding was monitored visually.

In order to perform PCR assays, the wells were instead filled with 10% sucrose solution. Sugar water was removed from the mosquito cages around 6–18 h prior to the biting tests. The chips were then placed on top of a mosquito cage. A strip of tape was adhered on top of the cage and the chip was placed such that some rows were placed atop the tape and were thus inaccessible for the mosquitoes to bite through. A heat source (resistive heater or tissue culture flask with warm water) was placed atop the chips such that their temperature was approximately 37 ^∘^C. Mosquitoes bit the chips for approximately 20–45 min, expectorating saliva in the process which was collected in the wells. A camera (Raspberry Pi camera module v2.1) attached to the bottom of the cage recorded mosquito activity on-chip. After approximately 20−45 min, the chips were removed from the cages. Chips bitten by ZIKV infected mosquitoes were placed on a hotplate set to 98 ^∘^C for 15 min to inactivate the virus and facilitate downstream processing.

PDMS base (Sylgard 184 part A) and crosslinker (Sylgard 184 part B) were mixed to a weight of 1 g and placed in a vacuum degasser for removing any bubbles. Approximately 0.75 g of the freshly prepared PDMS was added to a glass slide (75 mm × 50 mm). The PDMS was spread evenly using a pipette tip and was degassed for 2 min to remove any bubbles. The Vectorchip used for biting assay was then placed on the slide, membrane-side down into the thin layer of uncured PDMS carefully.

The chip was then dried either at room temperature overnight or in an oven at 80 ^∘^C for 20 min (for Zika-infected mosquitoes). This process adheres the chip to a solid glass slide by curing of the thin PDMS layer and allows the sugar water to evaporate in every well.

### RT-PCR assays

TaqMan™ Fast Virus 1-Step Master Mix (Thermofisher USA, product no:4444432) was used for the amplification assays. Primers and probes used for the amplification of *Aedes aegypti* mitochondrial DNA and Zika RNA have been listed in Supplementary Table 2.

The RT-PCR reaction mix was freshly prepared for each assay. For every 20 μL of RT-PCR reaction:

Fast virus mix - 5 μL

Fwd Primer - 1 μL

Rev Primer - 1 μL

Fluorescent probe - 0.5 μL

DI water - add as required to total 20 μL

Four μL of reaction mix was pipetted into all wells. Positive control wells were spiked with *Aedes aegypti* DNA or Zika virus RNA (0.5 μL each). Silicone oil (Sylgard 184 part A) was poured over the wells to prevent evaporation. The chips were placed at room temperature for 10 min to allow the viscous silicone oil enter the wells. If needed, gentle degassing (Lab vacuum line, vacuum measured as approximately 28 inches of Hg on gauge OR 40 Torr) for 15 min helped the oil enter the wells. The chips were then placed on a flat-plate thermocycler (GenePro, Bioer Tech.) and RT-PCR was performed for 42 cycles.

50 ^∘^C: 10 min

95 ^∘^C: 45 s

cycle × 42 (95 ^∘^C: 35 s; 60 ^∘^C: 80 s)

The chips were then scanned using a fluorescence image scanner (Typhoon FLA 9000, GE Healthcare) to obtain the images.

### Well-plate assay

Biting and PCR assays were performed on a 96 well plate in order to validate the RT-PCR efficiency in the chips. We loaded the wells with 2% ultra-low gelling temperature agarose (product code A2576, Sigma Aldrich, USA). The plate was placed at 4 ^∘^C for 30 min allowing the agarose to gel. Thereafter the plate was placed at 37 ^∘^C for 30 min, which warms up the plate (but does not melt the gel). A layer of parafilm was then stretched over the top of the plate to act as an artificial skin membrane. The plate was then placed upside down on top of the cage, with the parafilm touching the cage surface. Mosquitoes had access to the plate for approximately 30 min. The plate was then removed from the cage and the parafilm was carefully peeled off. Agarose gel was used as feeding media during this test to minimize the risk of cross-contamination between wells during the peeling off of the parafilm membrane. While liquid samples can leave droplets sticking to the parafilm, which could result in cross-contamination between wells, the gel medium is less likely to be displaced when the plate is placed upside down. The well plate was dried at room temperature overnight.n mix was added to the wells to perform qPCR tests on a real-time PCR system (StepOnePLus, Applied Biosystems). The reaction mix and thermocycling conditions were identical to as used for PCR assays in the Vectorchips.

### Focus forming assays

Chips were loaded with Dulbecco’s modified eagle medium (DMEM, Sigma Aldrich, USA, Cat. No. FG0435-500ML) supplemented with 10% fetal bovine serum (FBS, gibco, Life Technologies USA, Ref. 10438-018) in each well and placed over cages with approximately 50 female Mayaro virus-infected *Aedes aegypti* mosquitoes. The mosquitoes were allowed to bite the chips for 30 min. The negative control chips were bitten by uninfected mosquitoes. Chips were then removed from the cages and 10 μL of Vero cell suspension (1000 cells/μL) was dispensed into each well. To allow the virus particles to adsorb on to the cell surface, chips were incubated for one hour at 37 ^∘^C with 5% CO_2_. To remove unbound viral particles, cells were washed once with DMEM without FBS. Thereafter, 20 μL of 1% methylcellulose (MC) overlay medium supplemented with DMEM and 5% FBS was added to each well of the chips. Chips were then incubated at 37 ^∘^C incubator with 5% CO_2_ for 24 h. Note, to minimize the evaporation rate of culture media from the wells the chips were incubated in a humidified secondary container. The MC overlay medium was removed, and cells were fixed with 4% PFA for 20 min at room temperature (RT). After the removal of PFA, cells were washed with 1X PBS. Cell monolayers were then blocked for one hour in PBS with 3% BSA supplemented with 0.1% TritonX-100. After blocking, monolayers were incubated with CHIK-48 primary antibody that cross-react with Mayo virus E2 Envelope Glycoprotein (BEI resources; NR-44002 -1:500) overnight at 4 ^∘^C in blocking solution. Unbound primary antibodies were washed with PBS and incubated with Alexa-488-Conjugated secondary antibody (Invitrogen; A28175-1:500) for one hour at RT in PBS/3%BSA/0.1%TritonX-100. The secondary antibodies were removed and washed with PBS. Chips were imaged on an Olympus BX41 epifluorescent microscope, and foci were quantified.

### Biological samples

The following reagents were provided by the Centers for Disease Control and Prevention for distribution by BEI resources, NIAID, NIH: *Aedes aegypti*, Strain D2S3, Eggs, NR-45838. The following reagents were obtained through BEI Resources, NIAID, NIH: *Culex tarsalis*, Strain YOLO, Eggs, NR-43026, genomic RNA from Zika Virus, MR 766, NR-50085, and as part of the WRCEVA program: Mayaro Virus, BeAn343102, NR-49909. *Aedes aegypti* strain KPPTN, *Aedes albopictus* strain BP, and ZIKV strain FSS13025 were provided by Louis Lambrechts (Institut Pasteur). *Aedes aegypti* DNA was obtained using lab extraction from homogenized, dead mosquitoes.

### Mosquito rearing

A hatching broth was prepared by grinding 1 g of fish food granules (Aqueon goldfish granules) and mixing in 1 L of DI water. The solution was autoclaved and allowed to cool down to room temperature. The broth was then added to a plastic tray filled with 1 L DI water and eggs were hatched in the trays (200 eggs per tray). Ground fish food granules (Aqueon goldfish granules) were provided as feed for the larvae. The pupae were transferred to cups and placed in polypropylene rearing cages (Bugdorm, Taiwan). Mosquitoes were reared at 28 ^∘^C, 80% humidity with 12 h light-dark cycles. Flasks with sugar water (10%) and cotton wicks were placed in the rearing cages for the mosquitoes.

### Infectious blood meal

Approximately 55 *Aedes aegypti* females (11 days old) housed in cardboard containers were offered an infectious blood meal of defibrinated sheep blood supplemented with Zika virus at 1 × 10^7^ FFU/mL using a hemotek blood feeder. For FFA assays, *Aedes aegypti* females (11 days old) were offered a meal of whole human blood supplemented with Mayaro virus at 4 × 10^7^ FFU/mL using a glass feeder jacketed with 37 ^∘^C distilled water. Starved females were allowed to feed for 15−30 min and subsequently cold anesthetized to separate fed and non-fed individuals. Infected adults were maintained at 28 ^∘^C and 80% humidity having continuous access to a 10% sugar solution. Experiments were performed at 17 and 18 days post infection for Zika virus and 14 days post infection for Mayaro virus.

### Fluorescent saliva imaging

To facilitate imaging of fluorescent saliva, *Aedes aegypti* were allowed to feed on a solution containing 0.4% rhodamine B and 10% sucrose in water for at least 48 h. The rhodamine B ingested during sugar feeding stains the mosquito body including saliva^44^. Imaging was performed using a 532 nm, 5 mW laser (Sparkfun, COM-09906) for illumination, and a 550 nm longpass filter (Thorlabs FEL0550) as an emission filter. Images were acquired using a Basler acA2040-90um camera controlled using Pylon 5 software running on an Ubuntu 18.04 computer (NUC8i7BEH). The camera was equipped with a 100 mm macro lens (Canon macro EF 100 mm f/2.8L)^25^. Fluorescent saliva droplets on Vectorchips were quantified by scanning the chips before and after the biting assays on Typhoon FLA 9000 gel scanner. Salivary droplet diameter was analyzed using ImageJ version 1.53c. The droplets were assumed to be deposited as hemispheres on the membrane surface, and the volume of droplets was estimated using this assumption.

### Data analysis

Graphpad Prism 5 was used for creating the graphs. Images were analyzed using ImageJ version 1.53c.

### Reporting summary

Further information on research design is available in the Nature Research Reporting Summary linked to this article.

## Supplementary information

Supplementary Information File

Reporting Summary

Description of Additional Supplementary Files

Supplementary Movie 1

Supplementary Movie 2

Supplementary Movie 3

Supplementary Movie 4

Supplementary Movie 5

Supplementary Movie 6

Supplementary Movie 7

## Data Availability

Data generated or analyzed in this article are included in the published article and its Supplementary Files. Source data are provided with this paper.

## Source data

Source Data

## Code availability

Code used for data analysis in this article was written in Python 3 and is available on github. Image recognition algorithms used to track mosquito movement on the chip are available at https://github.com/felixhol/vectorChip. Algorithm for analytical analysis of distribution of bites on chip is available on github at https://github.com/Shailabhkr/Vectorchip.
