# Supplementary material for: A microfluidic platform for highly parallel bite by bite profiling of mosquito-borne pathogen transmission
Source: Nat Commun. 2021 Oct 14;12:6018. doi: 10.1038/s41467-021-26300-0 (PMC8516912; doi:10.1038/s41467-021-26300-0)
Supplement: Supplementary file 1 — Supplementary Information File [file 41467_2021_26300_MOESM1_ESM.pdf]

Supplementary Information

A microfluidic platform for highly parallel bite by bite profiling of mosquito-borne pathogen transmission

Shailabh Kumar†[1], Felix J. H. Hol†[1, 2, 3], Sujit Pujhari[4, 5], Clayton Ellington[1], Haripriya Vaidehi Narayanan[1], Hongquan Li[6], Jason L. Rasgon[4], Manu Prakash[1,*]

1 Department of Bioengineering, Stanford University, Stanford, CA, USA
2 Insect Virus Interactions Unit, Department of Virology, Institut Pasteur, Paris, France
3 Center for Research and Interdisciplinarity, U1284 INSERM, Université de Paris, Paris, France
4 Department of Entomology, The Center for Infectious Disease Dynamics, and the Huck Institutes of The Life Sciences, The Pennsylvania State University, University Park, PA, USA
5 Department of Pharmacology Physiology and Neuroscience, University of South Carolina School of Medicine, Columbia, South Carolina, USA
6 Department of Electrical Engineering, Stanford University, Stanford, CA, USA

† these authors contributed equally
* Corresponding author: manup@stanford.edu

Supplementary Note 1: A simple mathematical model for mosquito-chip interactions

We considered a simple mathematical model to generate a first approximation for the distribution of bites on a chip. We make the following assumptions:

a) Mosquitoes probe at a constant frequency (η bites/min),

b) Bites are independent event,

c) Mosquitoes bite one well at a time,

d) One well can only be bitten by one mosquito at a time. e) Here we do not account for spatial correlations of biting behavior of a single mosquito

We introduce the variables as N_m = Number of mosquitoes, N_w = Number of wells, t = total time of the experiment, k = number of bites a well receives in total time t, P = probability of getting k bites in time t, m = Number of total bites in time t, where m= $N_m \eta t$.

We develop a method to determine the distribution of these bites in N_w wells, based on the above mentioned parameters. There are total m bites that land on the chip. In order to find the probability of getting k bites on a single well, we start with finding the number of ways in which we can choose k out of m bites: ${}^{m}_{k}C = m!/(k! \times (m-k)!)$

Now, we consider the chance that k bites are on a single selected well $= 1/N_w^k$

And, the chance that rest of the bites are not on the selected well $= (1-1/N_w)^{m-k}$

Therefore, we can write the probability of obtaining k bites on a single well as

$$P[X = k] = m!/k!(m-k)! \times 1/N_w^k \times (1-1/N_w)^{m-k} \tag{1}$$

Replacing m by $N_m \eta t$ we get,

$$P[X=k] = N_m\eta t!/(k!(N_m\eta t-k)!) \times 1/N_w^k \times (1-1/N_w)^{N_m\eta t-k} \tag{2}$$

expanding the third term we get,

$$P[X=k] = N_m\eta t!/(k!(N_m\eta t-k)!) \times 1/(N_w \times (1-1/N_w))^k \times (1-1/N_w)^{N_m\eta t} \tag{3}$$

Equation 3 can be used to calculate the probability of obtaining k bites per well while changing the variable input. Figure S3 shows a range of plots obtained by changing the input variables in equation (4)– using numbers which might be expected in lab or field experiments.

We can further approximate the above equation by expanding the first term,

$$N_m\eta t!/(k!(N_m\eta t-k)!) = 1/k! \times 1/(N_m\eta t-k)! \times (N_m\eta t-k)! \times \{(N_m\eta t) \times (N_m\eta t-1) \times .....(N_m\eta t-k-1)\} \tag{4}$$

or,

$$N_m\eta t!/(k!(N_m\eta t-k)!) = 1/k! \times \{(N_m\eta t) \times (N_m\eta t-1) \times .....(N_m\eta t-k-1)\} \tag{5}$$

or we can approximate this as,

$$N_m\eta t!/(k!(N_m\eta t-k)!) \approx 1/k! \times \{(N_m\eta t)^k\} \tag{6}$$

Using equation (6) in equation (3) we get,

$$P[X=k] \approx 1/k! \times (N_m \eta t/(N_w-1))^k \times (1-1/N_w)^{N_m \eta t} \tag{7}$$

We can approximate this further as,

$$P[X=k] \approx e^{(-N_m \eta t/N_w)} \times (N_m \eta t/N_w)^k/k! \tag{8}$$

In this form, this equation represents a Poisson distribution of the form,

$$P[k] = e^{-\lambda} \times (\lambda)^k/k! \tag{9}$$

with the expected rate of events (*i.e.* bites per well), $\lambda = N_m \eta t/N_w$.

Supplementary Note 2: Feeding media optimization

The relationship between the feeding media and the mosquito biting behavior can be carefully explored to improve sample collection on *Vectorchip* [1], for both field surveillance as well as laboratory-based analysis of infectious pathogen transmission.

While blood-meals could be ideally suited for studying mosquito behavior on chips, they pose several inhibitory challenges for one-step RT-PCR as well as focus forming assays [2]. Hence, feeding media which allows sensitive biomolecular analysis while closely resembling blood meals can help encourage analysis of more physiologically relevant biting dynamics and pathogen transmission events. Mosquito neurons rely on sensing several molecules (glucose, $NaHCO_3$, salts, and ATP) in the feeding media to identify blood resulting in physiological responses such as prolonged feeding, abdominal engorgement, and direct delivery of food to the midgut [3]. We tested the performance of PCR on chips where sucrose was laced with these components prior to drying, followed by addition of PCR reaction mix and amplification. We observed weaker or no amplification

if components such as 100 mM NaCl, or 25 mM $NaHCO_3$ were added to the sucrose solution prior to amplification. We tested the PCR performance of sucrose spiked with 100 µM ATP or dNTP mix in *Vectorchip* and realized that they performed as well as DI water for amplification (Fig 9a) (n = 3). We selected the nucleotide concentration as 100 µM, as the PCR master mix usually contains nucleotides at similar concentrations, and hence this addition does not influence the amplification significantly. Furthermore previous reports have indicated 100 µM ATP to be a strong promoter of feeding by mosquitoes [3]. Therefore sucrose mixed with 100 µM ATP can be utilized as an improved feeding media for RT-PCR on *Vectorchip*.

Blood meal-mimic feeding on-chip is a highly desired target for analysis of viral transmission during bites. Significantly, DMEM already contains glucose (25 mM), NaCl (110 mM), and $NaHCO_3$ (44 mM) at concentrations ideally suited for identification by mosquito sensory neurons as blood-meal [3]. We comparatively evaluated the feeding response of mosquitoes using *Vectorchip* loaded with either blood or DMEM (supplemented with 10 % fetal bovine serum (FBS) and 1 mM ATP). We observed that the fraction of engorgements in sampled mosquitoes fed using DMEM were similar or better than obtained using blood in a duration of 45 minutes (n =3) (Fig 9b).

Our results indicate that compared to traditionally used salivary collection method such as forced salivation, feeding media in *Vectorchip* can be optimized for revealing physiologically relevant biting dynamics and viral transmission for further improved analysis of bite-based transmission events.

Supplemetary Note 3: *Vectorchip* cost analysis

Here we discuss current and potential future costs associated with application of high-throughput nucleic acid amplification in PDMS-based *Vectorchips*.

The cost of chip fabrication depends on cost of the silicone raw material, as well as processing infrastructure. This is further dependent on chip design and availability of local resources. We

used approximately 60 grams of PDMS (Sylgard 184 part A and part B at 10:1 ratio) to obtain 6 chips. The price of the raw material was approximately $120 for 450 grams of PDMS. Therefore, we can calculate that every chip cost $2 in raw materials to fabricate. We used shared facility and laboratory infrastructure (laser cutter and spin-coater) to fabricate the chips and it is difficult for us to estimate the cost for these steps. However, alternate low-cost methods to make through-holes in PDMS such as manual punching can also be used. In comparison, examples of cost of well plates typically used for PCR reactions are $2.30 per plate (Grenier, non-sterile, 96 wells, pack of 100, product id: Sigma M2936), or $4.74 per plate (Greiner, 384 wells, pack of 100, product id: Sigma M1686). Therefore, cost of manufacture for *Vectorchips* currently appears to be on the same order of magnitude as commercially available, bulk-manufactured products. We expect that the manufacturing price for *Vectorchips* can likely be further reduced through bulk purchase of raw material, change of raw material to lower-cost silicone rubber, and scale up during processing.

The cost per reaction depends on the price and volume of reagents used for PCR. As compared to a standard PCR reaction for a single homogenized mosquito, our reaction uses a 5-fold lower reaction volume and thus cost. We used the TaqMan™ Fast Virus 1-Step Master Mix (Thermofisher USA, product no:4444432, price $374 per mL). The cost per reaction can be reduced further by reducing the reaction volume by 1 or 2 orders of magnitude [4].

In order to perform thermocycling and fluorescence readout on a flat chip, we utilized a thermocycler with a flat-plate attachment and Typhoon gel scanner. These devices were used as they were conveniently available to us to rapidly demonstrate the principles of device operation. Diverse commercial as well as low cost open-source options have been realized for PCR instrumentation in the last couple decades [5–8], which can be utilized for field or laboratory use. Furthermore, we are working towards utilizing isothermal amplification methods and colorimetric readouts for detection of target nucleic acids in *Vectorchips* [9], which would further simplify the tools needed for amplification and detection.

References

[1] Marinotti, O., James, A. A. & Ribeiro, J. C. Diet and salivation in female aedes aegypti mosquitoes. *Journal of Insect Physiology* **36**, 545–548 (1990).

[2] Sidstedt, M. *et al.* Inhibition mechanisms of hemoglobin, immunoglobulin g, and whole blood in digital and real-time pcr. *Analytical and bioanalytical chemistry* **410**, 2569–2583 (2018).

[3] Jové, V. *et al.* Sensory discrimination of blood and floral nectar by aedes aegypti mosquitoes. *Neuron* **108**, 1163–1180 (2020).

[4] Beer, N. R. *et al.* On-chip single-copy real-time reverse-transcription pcr in isolated picoliter droplets. *Analytical chemistry* **80**, 1854–1858 (2008).

[5] Marx, V. Pcr heads into the field. *Nature methods* **12**, 393–397 (2015).

[6] Zhu, H., Yaglidere, O., Su, T.-W., Tseng, D. & Ozcan, A. Cost-effective and compact wide-field fluorescent imaging on a cell-phone. *Lab on a Chip* **11**, 315–322 (2011).

[7] Mendoza-Gallegos, R. A., Rios, A. & Garcia-Cordero, J. L. An affordable and portable thermocycler for real-time pcr made of 3d-printed parts and off-the-shelf electronics. *Analytical chemistry* **90**, 5563–5568 (2018).

[8] Lu, Q. *et al.* A modular, open-source, slide-scanning microscope for diagnostic applications in resource-constrained settings. *PloS one* **13**, e0194063 (2018).

[9] Li, E., Larson, A., Kothari, A. & Prakash, M. Handyfuge-lamp: low-cost and electricity-free centrifugation for isothermal sars-cov-2 detection in saliva. *medRxiv* (2020).

Supplementary Figures

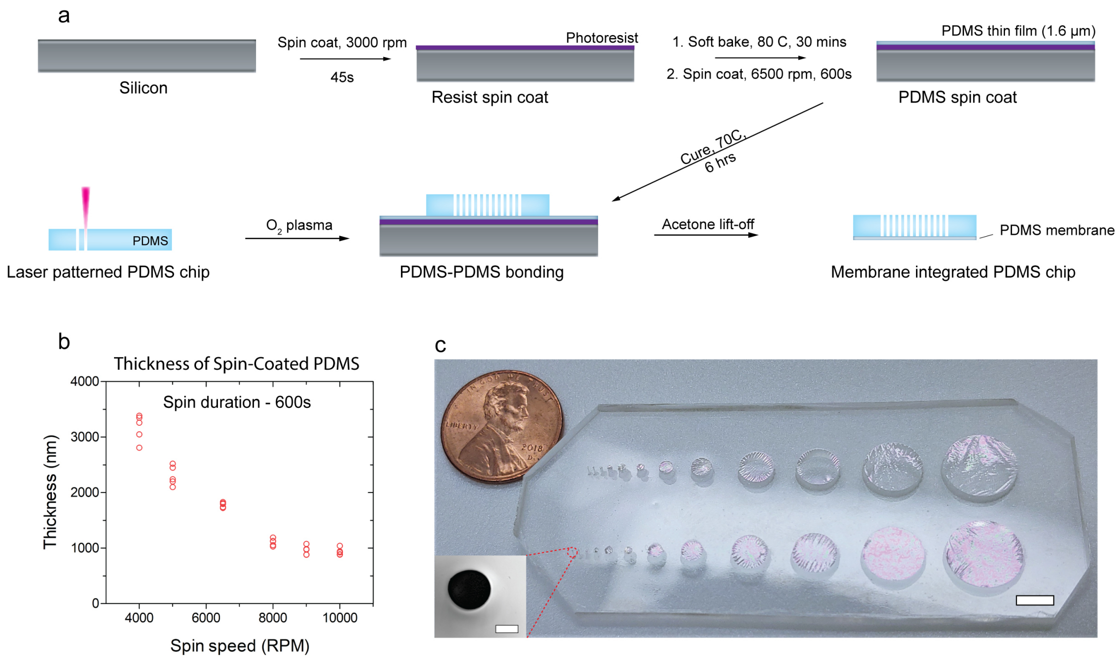

Supplementary figure 1: **Fabrication of *Vectorchips***. a) Schematic shows detailed steps for the fabrication of *Vectorchips*. Laser patterning and soft lithography were used for fabrication. A sacrificial resist layer was spun onto a flat silicon wafer. A 30 min soft-bake was performed to dry the resist layer. A thin PDMS film was spin-coated onto the resist layer and placed in an oven for curing. PDMS blocks (Sylgard 184, 10:1 base:crosslinker) were prepared in the lab and patterned using a CO_2 laser cutter. The patterned chips were bonded to the PDMS thin film using plasma oxidation. The wafers were placed in acetone where the resist layer was dissolved and the chips could be retrieved for cleaning and drying. b) Variation in thickness of the PDMS membrane with change in spin speed for a spin time of 600 seconds. The thickness was measured using a profilometer in lab. c) A chip showing close to 2 orders of magnitude variation in well and membrane size. The smallest wells were 150 µm in diameter whereas the largest were 1 cm. Scale bars represent 5 mm in (c) and 100 µm in (c, inset).

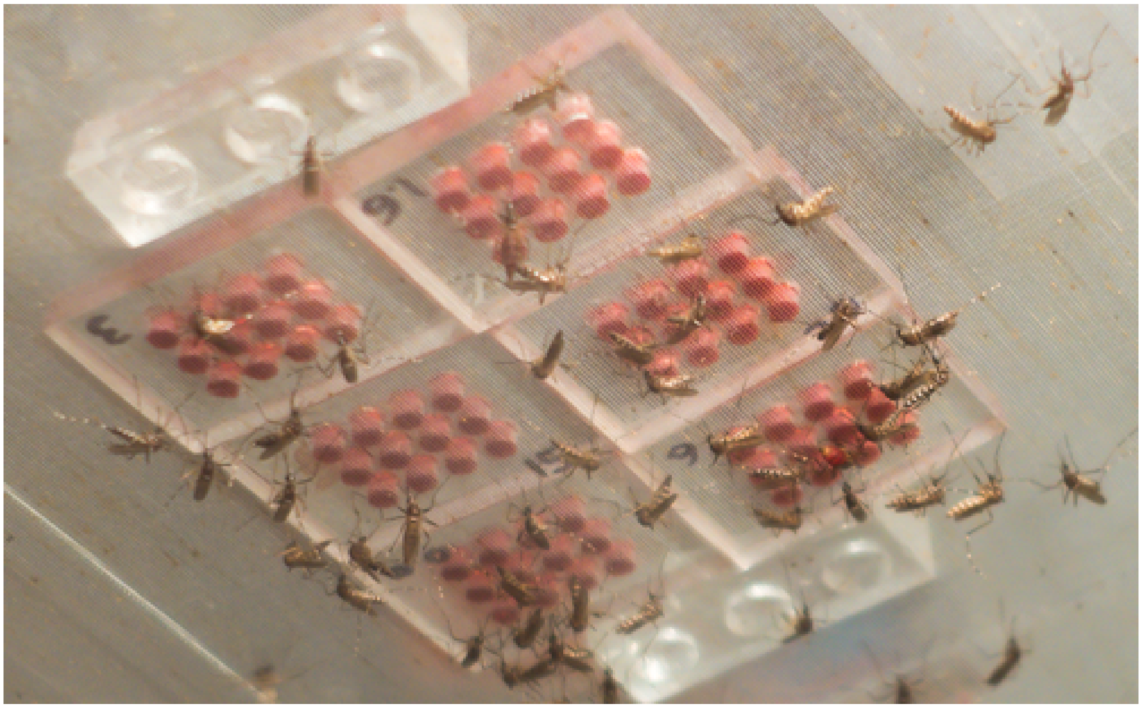

Supplementary figure 2: **Blood feeding on chips.** *Aedes aegypti* mosquitoes blood feeding from small chips with thin PDMS membranes. A resistive heater was placed on top of the chips to warm them and attract mosquitoes.

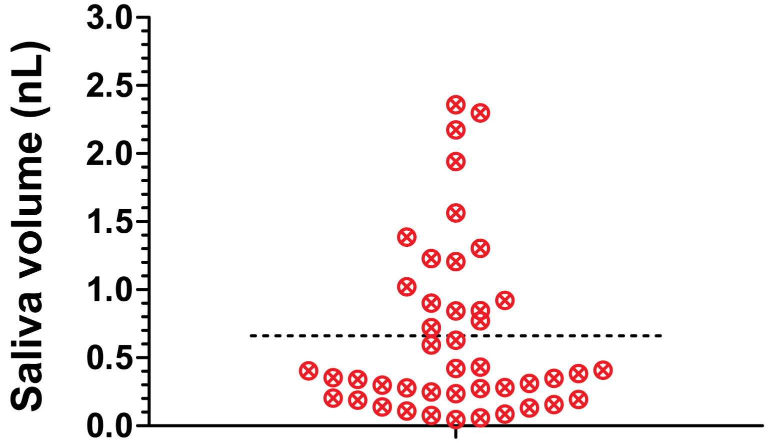

Supplementary figure 3: **Saliva volume estimate.** We estimated volume of salivary droplets released by probing *Aedes aegypti* mosquitoes as shown in Figure 3e,f. We analyzed image sequences identifying 44 deposited droplets and measured the diameter of these droplets. We assumed that the droplets deposited on the membrane interface are hemispherical to estimate the respective volumes. The mean volume of droplets was obtained as 0.66 nL. Source data are provided as a Source Data file.

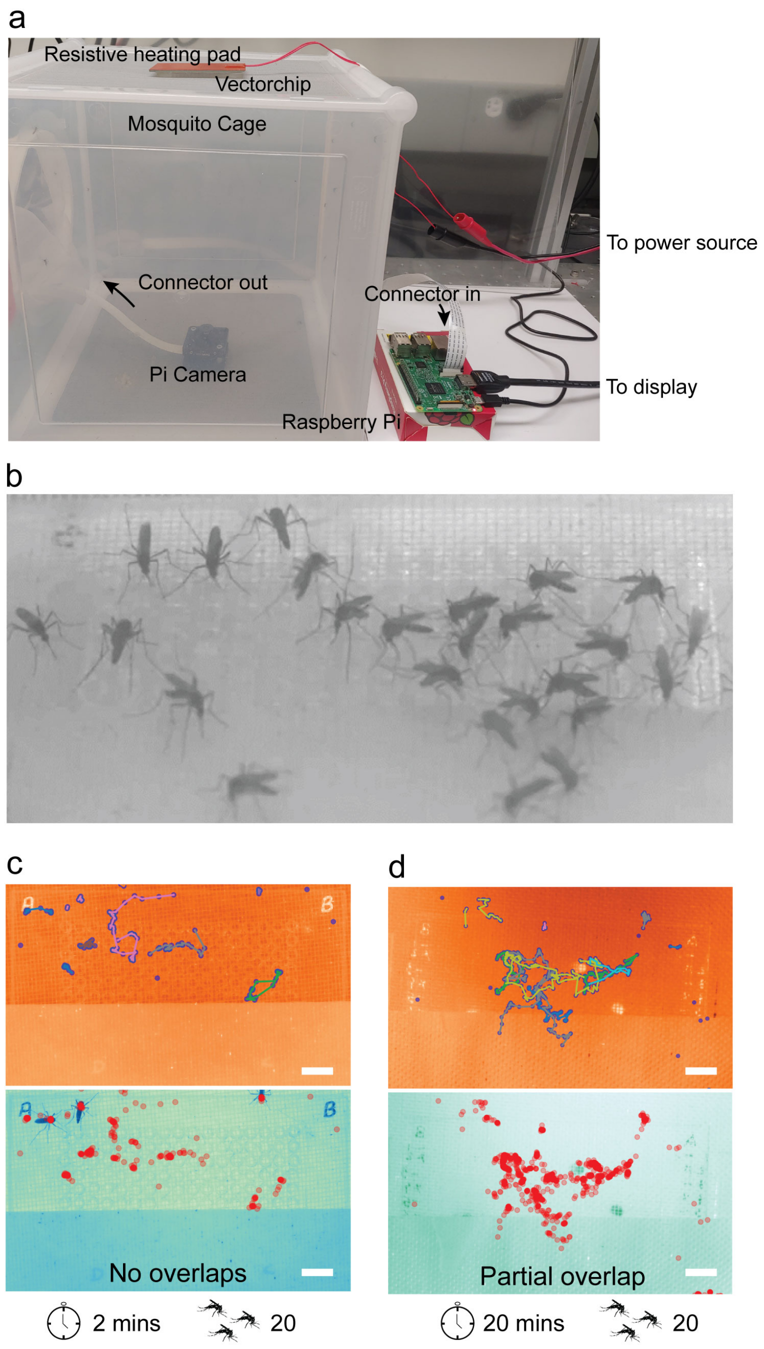

Supplementary figure 4: **Tracking on-chip biting.** a) Image showing setup for tracking mosquito movement during bite assays. A Raspberry pi camera was placed at the bottom of the cage and mosquitoes are allowed to access the sugar water through the mesh barrier at the top of the cage. A resistive pad was used as the source of heat to attract the mosquitoes. b) Mosquitoes drinking sucrose solution from a *Vectorchip*. (c, d) Tracking data showing two cases where no overlap or partial overlap between mosquito activity was observed dependent on the time of mosquito-chip interaction. Regions with no overlap in trajectories can provide statistical information about probing performed by individual mosquitoes. Symbols indicate duration of experiments and number of mosquitoes.

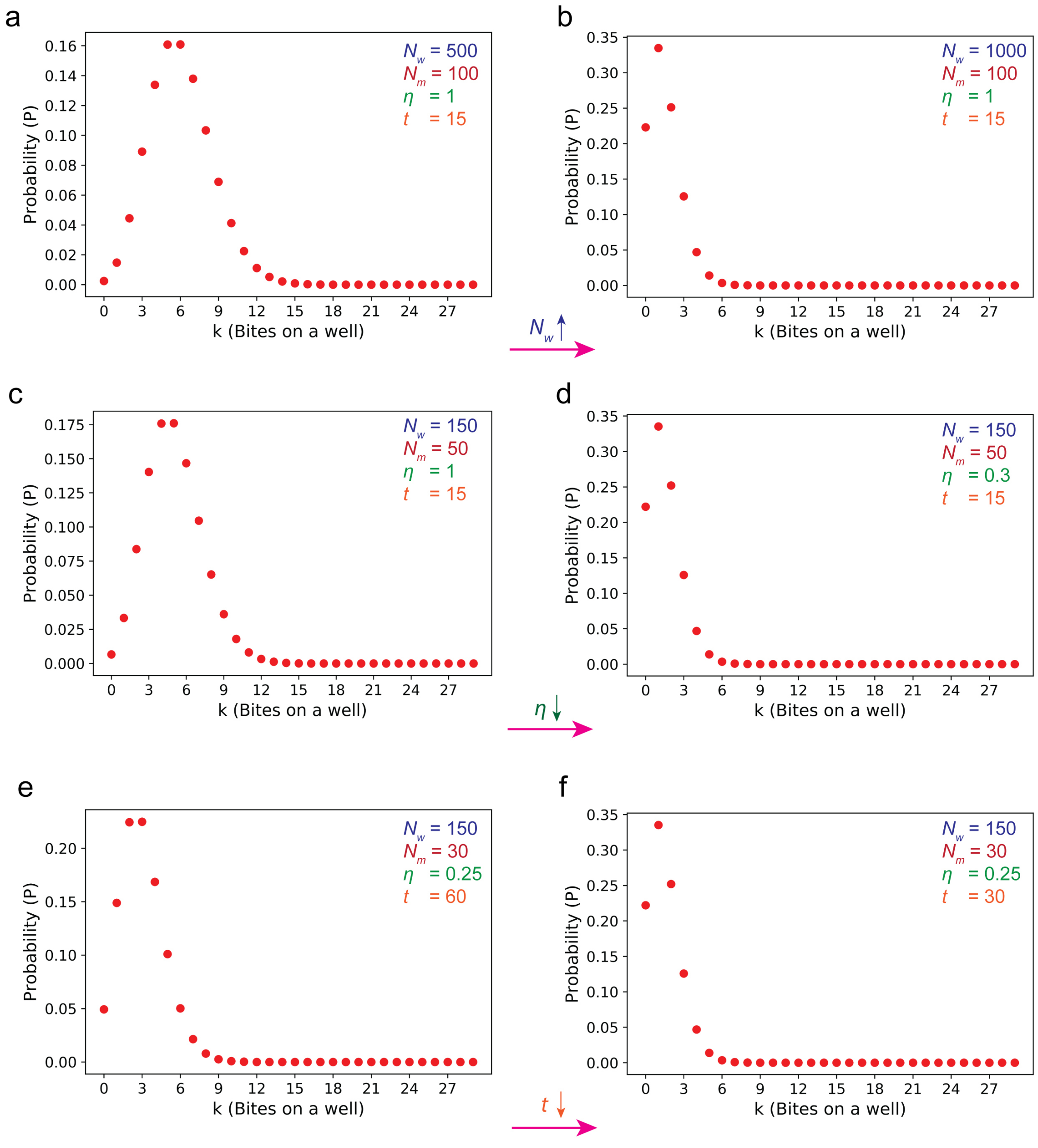

Supplementary figure 5: **Analytical model derived distribution of mosquito bites on chip.** We defined the number of mosquitoes = N_m, the number of wells = N_w, probing frequency = η. We estimated the probability (P) that a well receives k bites in given time t with variation in (a,b) ratio of available wells to number of mosquitoes, (c,d) probing frequency of a mosquito, and (e,f) time of experiment. The plots derived from equation 3 follow Poisson statistics for distribution of bites.

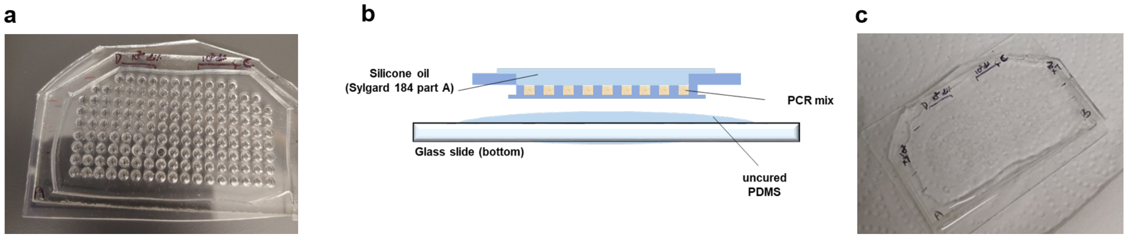

Supplementary figure 6: **PCR on-chip.** a) PCR reaction mix was pipetted into all the wells (volume - 4 µL per well) b) The top and bottom of the chip were covered with silicone oil to prevent evaporation. The bottom silicone was a 10:1 mix of Sylgard 184 base:crosslinker and helps the chip adhere to the glassslide. The top silicone oil was Sylgard 184 base (without crosslinker). c) Chip with PCR reaction mix and silicone oil layers prior to PCR.

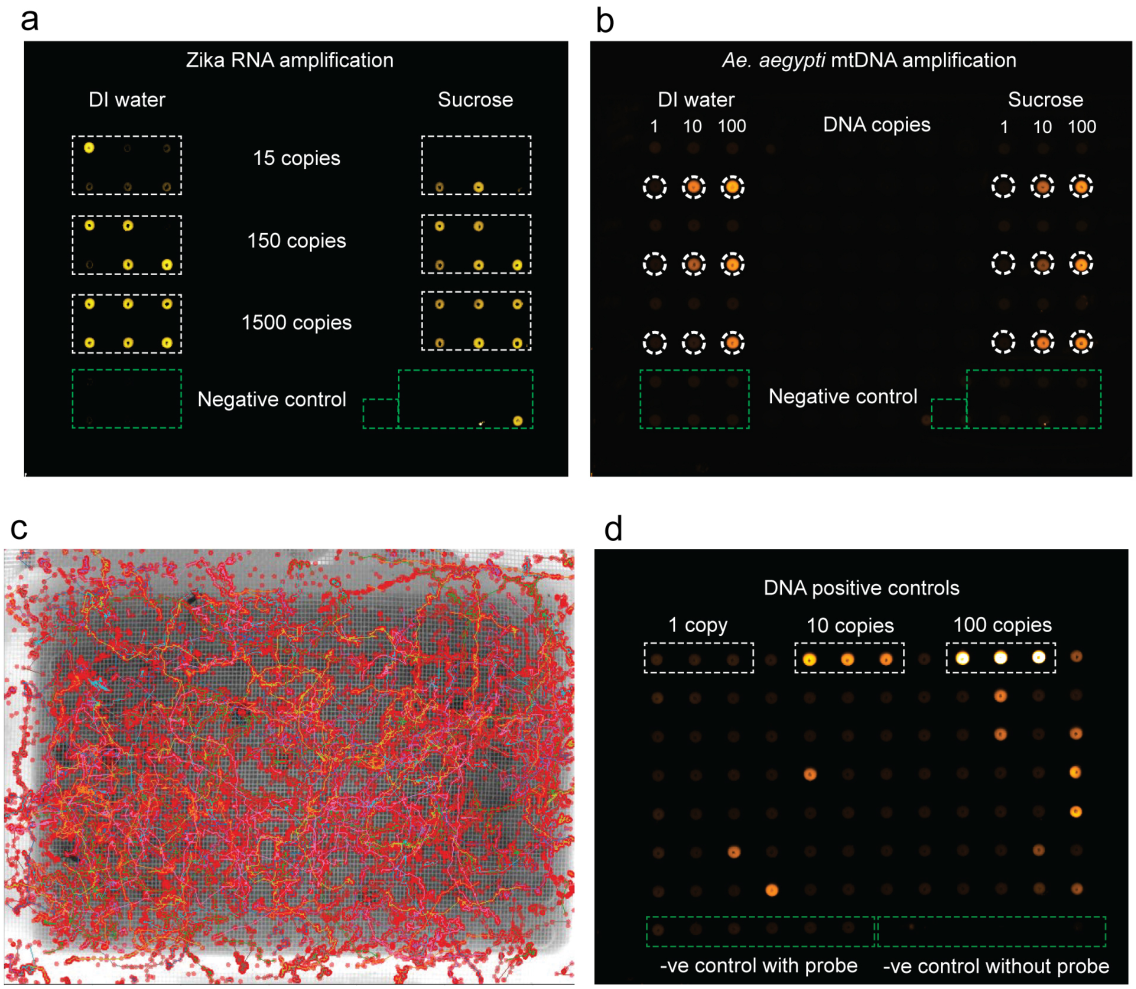

Supplementary figure 7: **PCR assays on 96 well plates.** PCR assays with manually spiked (a) RNA and (b) DNA was performed on the same 96 well plate for 42 cycles (4 µL reaction volume). The top row of each group of outlined wells shown in the figure (a) contained just RNA. The bottom rows of each group of outlined wells in (a) contained both DNA and RNA. The reactions were performed in both DI water and 10% sucrose to test if sucrose inhibits the reactions. Wells with amplification show increased fluorescence and are visible in the images. Amplification was observed in both cases for RNA (15-1500 copies) and DNA (10-100 copies). 1 false positive was observed for RNA amplification. (c, d) 96 well plate covered with parafilm was used to simulate the *Vectorchips* experiments. (c) On-chip tracking revealed mosquito translocation covering all regions of the 96 well plate. (d) PCR assays demonstrate detection of *Aedes aegypti* mitochondrial DNA in $\sim$ 17% of the available wells.

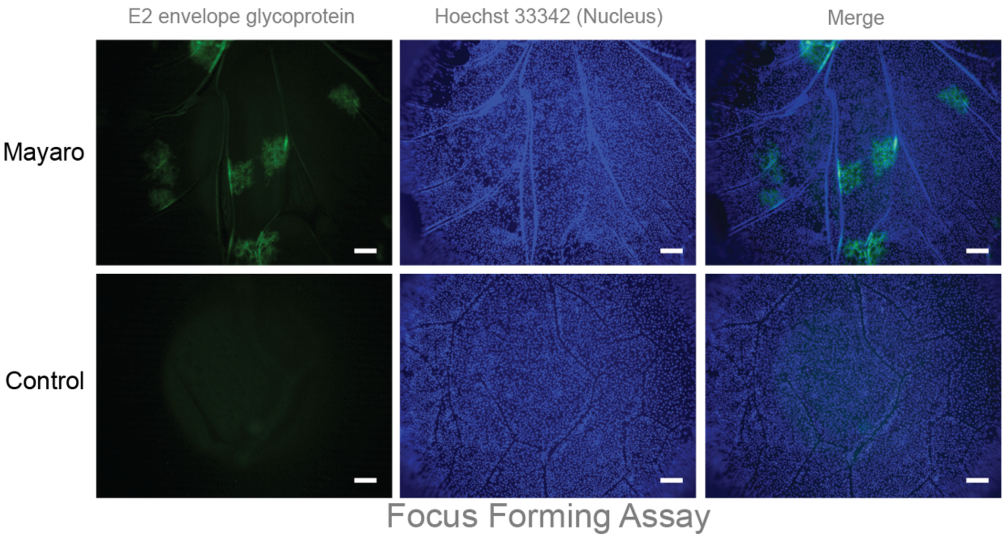

Supplementary figure 8: **Focus Forming Assays (FFA) on chip.** Mayaro viral particles were manually pipetted into sample wells in *Vectorchips*. A monolayer of vero cells were grown on the skin-mimic PDMS membrane (labeled using Hoechst 33342, nuclear stain). The Mayaro viral particles infected the cell monolayer resulting in foci and were visualized using a labeled antibody against the E2 envelope glycoprotein. The scale bar is 100 µm. The FFA experiment was replicated in 3 wells for both control and Mayaro virus samples (n = 3).

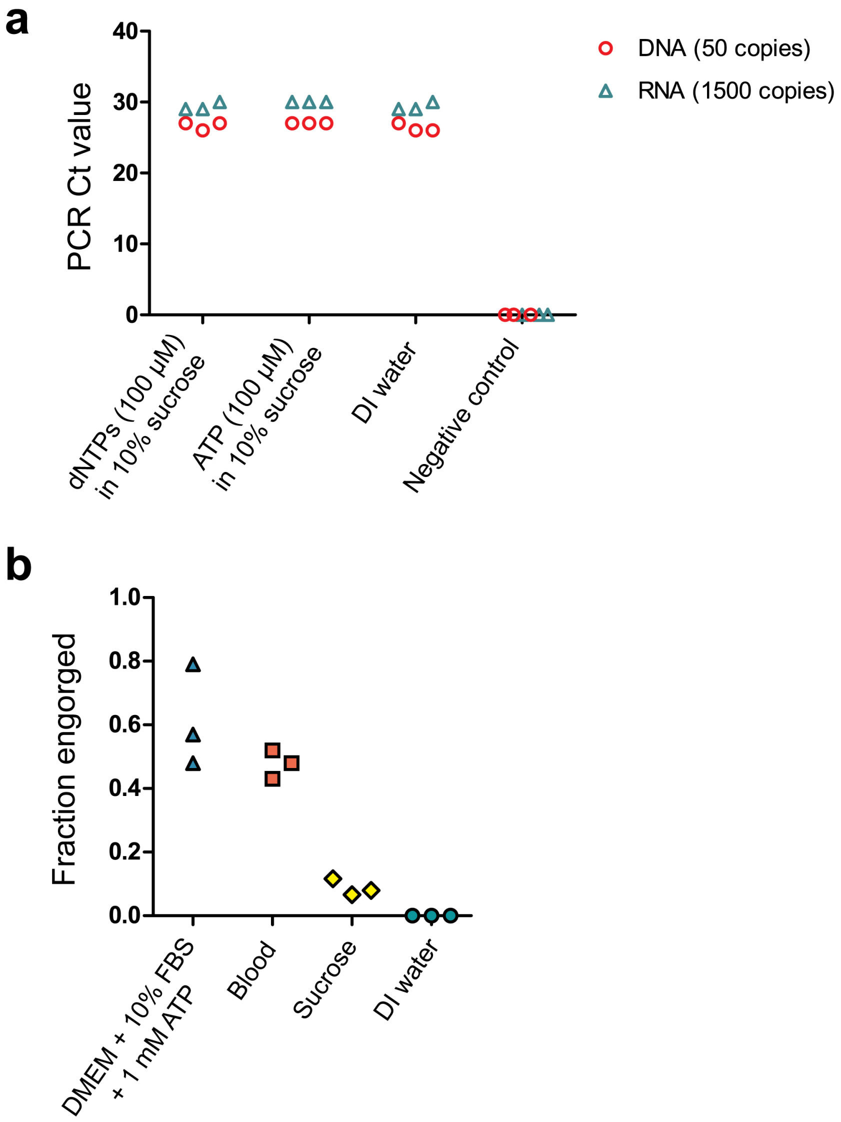

Supplementary figure 9: **Optimization of feeding media.** a) We added 100 µM ATP or dNTP mix to sucrose in *Vectorchips*. The samples were allowed to dry (replicating the steps of PCR assays performed after bites). PCR reaction mix was added to the wells to test nucleic acid amplification. Nucleic acid amplifications did not appear to be affected by addition of ATP or dNTP mix in the feeding media. This is indicated by the similar PCR cycle threshold (cT) values obtained for these solutions (n = 3) b)*Vectorchips* loaded with DMEM (supplemented with 10 % FBS and 1 mM ATP) showed on average higher fraction of mosquitoes with abdominal engorgement compared to mosquitoes fed on *Vectorchip* loaded with blood in a duration of 45 minutes. The feeding assays were repeated thrice for all samples (n =3) with a unique chip used per assay per sample (9 chips in total). Source data are provided as a Source Data file.

k (bites on a well)	Number of wells	Fraction of wells
0	82	0.8039216
1	15	0.1470588
2	4	0.03921569
3	2	0.01960784
4	0	0
5	1	0.009803922
6	0	0
7	0	0
8	0	0
9	0	0
10	0	0

Supplementary table 1: **Bites per well**. Table shows the number of bites per well quantified using the image shown in Figure 3h. This data was used to plot Figure 3j.

Oligonucelotide list	Sequence (5' -3')
Ae. aegypti mtDNA FWD Primer	ACACATGCAAATCACCCATTTC
Ae. aegypti mtDNA Rev Primer	CATTGGACAAGGCCTGTAACT
Ae. aegypti mtDNA Probe	HEX-AGCCCTTGA-ZEN-CCTTTAACAGGAGCT-3IABkFQ
Zika FWD Primer	CCGCTGCCCAACACAAG
Zika Rev Primer	CCACTAACGTTCTTTTGCAGACAT
Zika Probe	FAM-AGCCTACCT-ZEN-TGACAAGCAGTCAGACACTCAA-3IABkFQ

Supplementary table 2: **Primers and probes**. Table shows the list of oligonucelotides used for performing RT-PCR detection of *Aedes aegypti* DNA and Zika RNA.
