# Supplementary material for: A microfluidic platform for highly parallel bite by bite profiling of mosquito-borne pathogen transmission
Source: Nat Commun. 2021 Oct 14;12:6018. doi: 10.1038/s41467-021-26300-0 (PMC8516912; doi:10.1038/s41467-021-26300-0)
Supplement: Supplementary file 2 — Reporting Summary [file 41467_2021_26300_MOESM2_ESM.pdf]

nature research

Corresponding author(s): Manu Prakash

Last updated by author(s): Aug 30, 2021

Reporting Summary

Nature Research wishes to improve the reproducibility of the work that we publish. This form provides structure for consistency and transparency in reporting. For further information on Nature Research policies, see our Editorial Policies and the Editorial Policy Checklist.

Statistics

For all statistical analyses, confirm that the following items are present in the figure legend, table legend, main text, or Methods section.

n/a	Confirmed	
☐	☒	The exact sample size (n) for each experimental group/condition, given as a discrete number and unit of measurement
☐	☒	A statement on whether measurements were taken from distinct samples or whether the same sample was measured repeatedly
☒	☐	The statistical test(s) used AND whether they are one- or two-sided<br>*Only common tests should be described solely by name; describe more complex techniques in the Methods section.*
☒	☐	A description of all covariates tested
☐	☒	A description of any assumptions or corrections, such as tests of normality and adjustment for multiple comparisons
☒	☐	A full description of the statistical parameters including central tendency (e.g. means) or other basic estimates (e.g. regression coefficient) AND variation (e.g. standard deviation) or associated estimates of uncertainty (e.g. confidence intervals)
☒	☐	For null hypothesis testing, the test statistic (e.g. F, t, r) with confidence intervals, effect sizes, degrees of freedom and P value noted<br>*Give P values as exact values whenever suitable.*
☒	☐	For Bayesian analysis, information on the choice of priors and Markov chain Monte Carlo settings
☒	☐	For hierarchical and complex designs, identification of the appropriate level for tests and full reporting of outcomes
☒	☐	Estimates of effect sizes (e.g. Cohen's d, Pearson's r), indicating how they were calculated

Our web collection on statistics for biologists contains articles on many of the points above.

Software and code

Policy information about availability of computer code

Data collection	Image recognition algorithms were written in Python 3 see ref. \cite{hol2020biteoscope} for details, and used to track mosquito movement on the chip and are available on github (https://github.com/felixhol/vectorChip). Algorithm for analytical analysis of distribution of bites on chip is available on github at (https://github.com/Shailabhkr/Vectorchip).
Data analysis	Images were analyzed using ImageJ version 1.53c. Graphpad Prism 5 was used for creating the graphs and plotting the data.

For manuscripts utilizing custom algorithms or software that are central to the research but not yet described in published literature, software must be made available to editors and reviewers. We strongly encourage code deposition in a community repository (e.g. GitHub). See the Nature Research guidelines for submitting code & software for further information.

Data

Policy information about availability of data

All manuscripts must include a data availability statement. This statement should provide the following information, where applicable:

- Accession codes, unique identifiers, or web links for publicly available datasets
- A list of figures that have associated raw data
- A description of any restrictions on data availability

Data generated or analysed in this article are included in the published article and its supplementary information files. Source data file has been included with the submission.

Field-specific reporting

Please select the one below that is the best fit for your research. If you are not sure, read the appropriate sections before making your selection.

☒ Life sciences ☐ Behavioural & social sciences ☐ Ecological, evolutionary & environmental sciences

For a reference copy of the document with all sections, see nature.com/documents/nr-reporting-summary-flat.pdf

Life sciences study design

All studies must disclose on these points even when the disclosure is negative.

Sample size	We used the maximum number of mosquitoes available to us for the assays. This was dependent on the size of colony at the time of the assay. All experiments used a population size greater than 25 mosquitoes. This was a sufficiently large number of mosquitoes to bite the chips and deposit saliva, and was verified visually while performing the experiments. For all PCR and FFA assays a minimum sample size of three vectorchips was chosen, as this is considered to be statistically significant.
Data exclusions	No data were excluded from analyses.
Replication	All experiments were performed thrice independently to confirm replicability and were found to be replicable.
Randomization	Maximum number of available mosquitoes (which was dependent on size of colonies) were selected for the biting assays. For most of the assays the whole population underwent the same treatment (i.e. biting into vectorchips with identical feeding media). For assays where multiple groups were required (comparison of engorgements with feeding media), female mosquitoes was randomly distributed into multiple cages.
Blinding	Blinding was not performed or relevant. For most of the assays the whole population underwent the same treatment (i.e. biting into vectorchips with identical feeding media). For assays where multiple groups were required (comparison of engorgements with feeding media), female mosquitoes was randomly distributed into multiple cages. Experimenter had to visually assess the engorgements in all cases, and hence blinding was not relevant.

Reporting for specific materials, systems and methods

We require information from authors about some types of materials, experimental systems and methods used in many studies. Here, indicate whether each material, system or method listed is relevant to your study. If you are not sure if a list item applies to your research, read the appropriate section before selecting a response.

Materials & experimental systems

n/a	Involved in the study	
☐	☒	Antibodies
☐	☒	Eukaryotic cell lines
☒	☐	Palaeontology and archaeology
☐	☒	Animals and other organisms
☒	☐	Human research participants
☒	☐	Clinical data
☒	☐	Dual use research of concern

Methods

n/a	Involved in the study	
☒	☐	ChIP-seq
☒	☐	Flow cytometry
☒	☐	MRI-based neuroimaging

Antibodies

Antibodies used	1) CHIK-48 primary antibody (BEI resources; NR-44002 -1:500) 2) Alexa-488-Conjugated secondary antibody (Invitrogen; A28175-1:500)
Validation	CHK-48 antibody (NR-44002) : Anti-Chikungunya Virus E2 Envelope Glycoprotein raised in mice by MS Diamond group and distributed through BEI resources. According to BEI literature, this antibody cross-reacts with Mayaro virus. Rasgon lab has cross verified the specificity of the antibody for the Mayaro virus. (Brustolin et al, PLoS Negl Trop Dis. 2018 Nov 7;12(11):e0006895; Pujhari et al. BioRxiv NoV 2020 https://doi.org/10.1101/2020.11.18.388884)

Eukaryotic cell lines

Policy information about cell lines

Cell line source(s)	Vero (ATCC, CCL-81): ordered in June 2019.

Authentication	The cell line was not authenticated.
Mycoplasma contamination	Mycoplasma Negative (source: ATCC)
Commonly misidentified lines (See ICLAC register)	No commonly misidentified cell lines were used with the study.

Animals and other organisms

Policy information about studies involving animals; ARRIVE guidelines recommended for reporting animal research

Laboratory animals	Aedes aegypti, Strain D2S3, female, age 15-20 days were used for blood feeding assays, while age 17-25 days were used for uninfected PCR assays. Aedes aegypti, strain KPPTN, female, age 27-29 days were used for infected PCR and focus forming assays. Aedes albopictus, Strain BP, female, age 15-20 days were used for feeding assays. Culex tarsalis, Strain YOLO, female, age 15 -22 days were used for uninfected blood feeding assays.
Wild animals	Study did not involve wild animals.
Field-collected samples	Study did not involve field-collected samples.
Ethics oversight	No ethical approval was required for mosquito assays, as no human or animal subjects were involved.

Note that full information on the approval of the study protocol must also be provided in the manuscript.
