# Supplementary material for: A microfluidic platform for highly parallel bite by bite profiling of mosquito-borne pathogen transmission
Source: Nat Commun. 2021 Oct 14;12:6018. doi: 10.1038/s41467-021-26300-0 (PMC8516912; doi:10.1038/s41467-021-26300-0)
Supplement: Supplementary file 3 — Description of Additional Supplementary Files [file 41467_2021_26300_MOESM3_ESM.pdf]

Supplementary Movie 1: An *Aedes aegypti* female mosquito biting through 1.6 µm thick Vectorchip membrane (bottom-up view).

Supplementary Movie 2: An *Aedes aegypti* female mosquito biting through 1.6 µm thick Vectorchip membrane (side view).

Supplementary Movie 3: *Aedes aegypti* mosquitoes blood feeding on Vectorchips.

Supplementary Movie 4: *Aedes aegypti* female mosquito failing to bite through 40 µm thick Vectorchip membrane (side view).

Supplementary Movie 5: *Aedes aegypti* female mosquito failing to bite through 40 µm thick Vectorchip membrane (bottom-up view).

Supplementary Movie 6: Rhodamine-fed mosquitoes expectorating fluorescent saliva while probing.

Supplementary Movie 7: Tracking mosquito movement on-chip.
